# Enigmatic HCl detections as a potential signature of igneous activity on Mars

**DOI:** 10.1126/sciadv.aeb9422

**Published:** 2026-11-04

**Authors:** Alexander Trokhimovskiy, Daniel Mège, Oleg Korablev, Kevin S. Olsen, Anna A. Fedorova, Benjamin Taysum, Pierre-Antoine Tesson, Mikhail Luginin, Franck Montmessin, Pavel Vlasov, Alexey Grigoriev, Juan Alday, Denis Belyaev, Nikolay Ignatiev, Andrey Patrakeev, Nikita Kokonkov, Yuriy Ivanov, Joanna Gurgurewicz, Antoine Pommerol, Gabriele Cremonese, Nicolas Thomas

**Affiliations:** ^1^ Space Research Institute RAS, Profsoyuznaya 84/32, Moscow 117997, Russia.; ^2^ Centrum Badań Kosmicznych Polskiej Akademii Nauk (CBK PAN), Bartycka 18A, 00-716 Warszawa, Poland.; ^3^ Physikalisches Institut, Universität Bern, Bern, Switzerland.; ^4^ Department of Physics, University of Oxford, Oxford OX1 3PU, UK.; ^5^ German Aerospace Center (DLR), Institute of Planetary Research, Berlin, Germany.; ^6^ LATMOS/CNRS, Université Paris-Saclay, Sorbonne Université, Guyancourt, France.; ^7^ Research School of Astronomy and Astrophysics, Australian National University, Canberra, Australia.; ^8^ School of Physical Sciences, The Open University, Milton Keynes, UK.; ^9^ Instituto de Astrofisica de Andalucia, Granada, Spain.; ^10^ Main Astronomical Observatory (MAO), National Academy of Sciences of Ukraine, Kiev, Ukraine.; ^11^ Astronomical Observatory of Padova, INAF, Padova, Italy.

## Abstract

Mars is reputed to lack ongoing volcanic activity. However, marsquakes were revealed recently, and clues to volcanism younger than tens of million years have accumulated. While sulfur dioxide, so far undetected, could testify to ongoing eruptive activity, evidence of active magma chambers may be better identified by seepage of halogen gases, such as hydrogen chloride (HCl). Observations by Atmospheric Chemistry Suite led to the widespread observations of HCl at part-per-billion levels in the atmosphere of Mars during the warm perihelion period, attributed to aerosol-atmospheric sources. Here, we report HCl detections during the cold aphelion season that are isolated in the Alba Mons region. We also identify fresh and narrow (<1 meter) fissures in this area, which we interpret to be potential HCl pathways rooted into magmatic bodies at depth. Our results are best explained by ongoing igneous processes in the Martian interior.

## INTRODUCTION

On Mars, as on Earth, the chlorine cycle initiated by volcanic degassing from the deep interior upon planetary accretion (*1*, *2*) was fed by the subsequent volcanic eruptions, and, because of its high mobility and solubility, chlorine has been continuously recycled in water, sediments (*1*), permafrost (*3*), and ice clouds (*4*). However, contrary to Earth, the absence of recycling due to plate tectonics probably makes the volcanic source the main input to the surface-atmosphere system on Mars. The topic addressed here is is volcanism still contributing to this cycle on Mars today?

Mars exhibits substantial past volcanic activity, particularly during the Hesperian era (3.7 to 3 billion years ago) when huge lava plains were formed and the Amazonian era (less than 3 billion years ago) when the largest volcanoes in the Solar System were constructed. There is increasing evidence pointing to the presence of geologically recent volcanic and seismic events on Mars (*5*–*7*). Crater retention age calculations indicate that volcanic activity in the Tharsis region, including the vast Tharsis volcanic bulge, has persisted throughout the Amazonian period, with some lava flows and tephra eruptions dated to just a few tens of millions of years ago (*8*–*10*). Similar ages have been calculated for regions such as Elysium Planitia and Cerberus Fossae, where volcanic activity may still be ongoing (*11*). This activity might be linked to some of the seismic events detected by the SEIS experiment on the InSight lander (*12*).

Modern models of Mars’ interior structure and thermal state propose that mantle plumes could still be producing melt beneath the Tharsis region up to the present day (*13*). These findings indicate that Mars has retained enough internal heat to support volcanic activity until recently and possibly still does (*14*). However, efforts to detect active volcanism through thermal signatures (*15*, *16*) or surface manifestations (*11*) have yielded negative or inconclusive results.

Volcanic outgassing, which could alter the atmospheric composition, was also investigated. Among the potential volcanic gases, sulfur compounds such as SO_2_ and its dissociation products (SO, OCS, and H_2_S) received primary attention. Nevertheless, these compounds have not been detected within the current upper detection limits for SO_2_, ranging from 0.3 parts per billion by volume (ppbv) globally to 3 ppbv above volcanic regions (*17*–*19*). The absence of these detections should not be interpreted as evidence of Martian volcanic inactivity, as, even on Earth, the SO_2_ is not consistently degassed in every geodynamic environment (*20*, *21*).

Hydrogen chloride (HCl) is generally less abundant than SO_2_ in volcanic emissions but can sometimes be found in comparable or higher amounts in terrestrial fumaroles (*22*, *23*). Studies have shown that SO_2_ emissions are inversely correlated with HCl emissions and are particularly low in posteruptive periods. HCl, due to its solubility in magma, continues to be released even after volcanic activity has ceased (see section S10). In addition, Cl/H_2_O and Cl/S ratios in Martian meteorites and analyzed rocks and soils are elevated compared to Earth (*24*–*26*). This supports the hypothesis that HCl is among the most abundant volcanic gases on Mars (*27*).

In this article, we describe sensitive detections of HCl made by Atmospheric Chemistry Suite (ACS) solar occultations during the aphelion season above the Alba Mons region on Mars, in the northern Tharsis plateau. We interpret these results as potential dormant volcanism, probably interacting with permafrost while being released into the atmosphere.

## RESULTS

In 2020, a seasonal signal of HCl in the Martian atmosphere was detected (*28*) using the ACS (*29*) on the ExoMars Trace Gas Orbiter (TGO). Regular ACS observations began in spring 2018, mid-Mars year (MY) 34. Previous ground-based attempts to detect HCl had been unsuccessful, providing only upper limits in the parts per billion range (*30*–*32*). Those measurements turned out to have been taken during HCl-free seasons, as TGO observations over three Martian years have shown a distinct seasonal HCl cycle, with peak volume mixing ratios up to 4 to 5 ppbv occurring during the southern hemisphere spring and summer, between solar longitudes (Ls) of 230° and 330° (*4*, *28*, *33*–*35*). This season on Mars is marked by increased dust, higher temperatures, and elevated water vapor (*36*, *37*).

The absence of sulfur compounds at part-per-billion levels (*17*, *18*) and the widespread distribution of HCl during the southern summer suggest a nonvolcanic origin for the gas but rather its production from chlorine-bearing aerosols (*28*). The chlorine isotopic ratio was found (*38*) to be consistent with Martian soil values (*39*), supporting the link between HCl and chlorine-bearing aerosols. Gas-phase models could not explain the HCl seasonality, but models considering H_2_O photolysis products reacting with dust and ice aerosols are able to reproduce the observed HCl abundances, seasonal trend, and some features of its perihelion distribution, balancing the heterogeneous production and loss (*40*–*42*). Both processes are strongly temperature dependent and connected with water abundance, which explains exceptional HCl observations during the warm perihelion period and notable similarity with the water vapor vertical distribution (*33*–*35*, *43*).

This article presents localized HCl detections made during the opposing season near aphelion (Ls = 71°) period at the beginning of northern summer. The TGO science operations began in MY 34 postaphelion. Two detections during MY 35 northern summer around Ls = 110° close to Alba Mons (near 40°N latitude and 110°W longitude) were inconclusive because of limited data (*34*); then, the MY 36 season was not covered by observations. In MY 37, we carried out a dedicated search for HCl during this time period and geographic region, resulting in six HCl detections, mainly northeast to Alba Mons. Four detections occurred at Ls = 107° to 111° at high latitudes (55°N to 66°N), and two more occurred at Ls = 117° to 119° at lower latitudes (43°N to 48°N), as shown in Fig. 1A (also see table S1 and fig. S1). All eight detections from the two MY fall into a narrow longitude range of 96°W to 117°W. The suboccultation spots of three detections below 50°N latitude have nearly zero elevation, while the remaining five detections are located above −5 to −2 km with respect to the areoid (analogous to the “sea level” concept), which is typical for these latitudes.

**Fig. 1.**
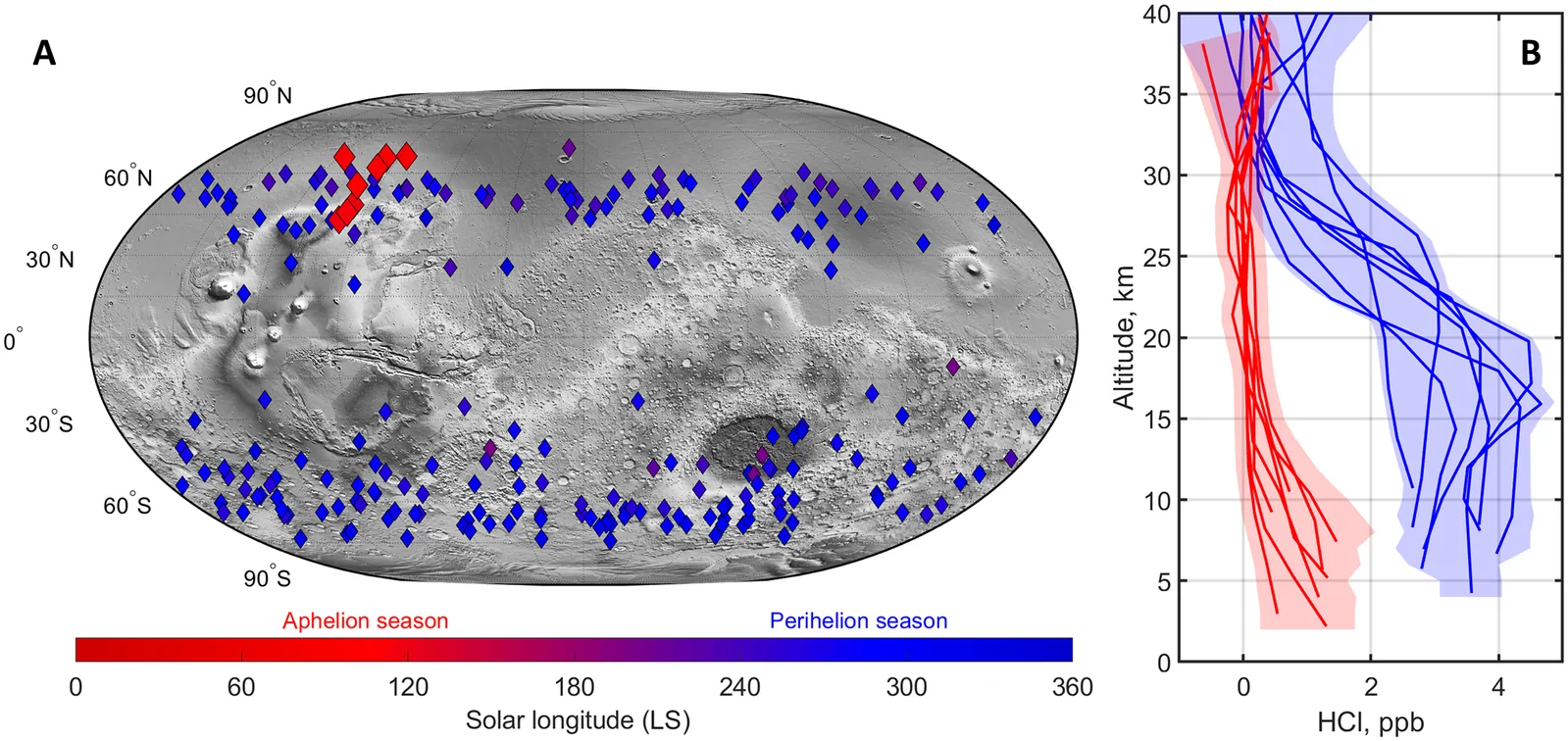
ACS observations of HCl in the Martian atmosphere. (**A**) Detections of hydrogen chloride in the Martian atmosphere at different seasons. The blue diamonds stand for globally distributed perihelion observations, while the red ones are isolated aphelion detections considered in this paper. (**B**) Vertical profiles of the HCl as measured by ACS MIR during the occultations. Color code indicates the season, as in (A); the shades represent the uncertainties of the retrievals. The family of typical perihelion HCl profiles (blue) was measured during southern hemisphere summer at latitudes from −66°S to −53°S, Ls = 292° to 304°, and MY 35.

During MY 35 and MY 37, at Ls = 100° to 120°, ACS observed 78 solar occultations in the northern hemisphere. Reduced aerosol optical thickness allowed for probing the lowermost atmosphere. Eight detections showed confident HCl signatures, while other observations over the planet including a few at the Alba Mons region indicated only sub–part per billion upper limits of ≤0.2 ppbv down to the low altitudes. When observed, HCl was confined to the lowermost layers, with a steep gradient in the first 10 km and maximum abundances up to 1.5 ppbv. The most recent observations during MY 38 aphelion (terrestrial summer 2025) also revealed HCl detections confined to the Alba Mons region; detailed analyses will be presented elsewhere.

Strongly localized in space and time, aphelion HCl detections, amidst an otherwise absence of HCl at that season on Mars, exhibit marked contrast with prolonged and widespread detections at perihelion. Hydrogen chloride abundances in both seasons are of the same order, although atmospheric conditions are starkly different: The northern hemisphere summer is on average 30 K colder than the southern, water vapor is confined to low altitudes, and there is little dust in the atmosphere. The intermittent character of aphelion HCl and the confinement of the observed profiles to the surface suggests that the gas is rapidly removed from the atmosphere, presumably by ice aerosols (*4*), which are nearly ubiquitous in the atmosphere above the hygropause (*44*, *45*). This mechanism is implemented in current atmospheric modeling schemes (*40*–*42*). The timing of the detections (Ls = 107° to 118°) coincides with global seasonal warming in the northern hemisphere, which has been demonstrated to reduce ice load (*36*), allowing higher abundances of HCl to develop in the atmosphere. This explains the seasonality of detections, but not their localization, since no evidence of any notable local warming around the area of interest has been found and water ice is still observed in the atmosphere (see section S9 and figs. S2 and S3).

The various pathways of atmospheric HCl production included in current chemistry models are found to be incompatible with our aphelion observations in terms of both HCl abundance and distribution. Negligible levels were predicted for Ls = 70°N and 40°N if HCl is produced by H atoms that react with FeCl_3_ (*40*). Other studies implementing heterogeneous reactions predict ppbv concentrations of HCl all over the north polar region at either low (*42*) or high altitudes (*41*), which are not confirmed by the ACS multiannual survey. Moreover, during this season, a prominent wave-3 pattern is known to develop in the Northern Hemisphere (*46*), driving the water vapor—assumed to correlate with the HCl production—to be concentrated in three corridors around longitudes of 150°W, 30°W, and 90°E but not over the Alba Mons region. There are therefore no observational or modeling indications of any atmospheric phenomena that explain the HCl enhancement north of Alba Mons (see also section S9 and figs. S2 and S3).

Three possible nonatmospheric causes may then be advocated: volcanism, mud volcanism (*47*), and decomposition of simple chloride compounds, such as perchlorate (*48*). A volcanic or hydrothermal source is supported by models of planetary accretion, which propose that HCl has been one of the most abundant volcanic gases on Mars (*27*), reflected in chlorine enrichment of Martian basalts (*24*). Mud volcanism, whose evidence was found north of Alba Mons (*49*), could outgas HCl if the mud interacts with a magma source (*50*), pointing again to a volcanic origin, or with an evaporitic host rock (*51*). Perchlorates were identified 140-km north of the northernmost HCl detection at the Phoenix landing site (*52*). However, both mud volcanism and perchlorates are thought to be pervasive in the northern lowlands (*47*, *53*, *54*) and cannot explain the localized nature of the HCl aphelion detections. It follows that a local, igneous source may best explain the ACS measurements reported here.

The highlands adjacent to the Alba Mons volcano exhibit a geological history of volcanism spanning billions of years, as evidenced by crater retention age determinations ranging from 3.4 billion years (*55*) to a few 10 million years (*10*, *56*). This extended period of activity suggests the potential for ongoing volcanic processes. It was also argued (*57*, *58*) that volcanic activity in the Tharsis region has migrated northward over time, which aligns with the presence of active igneous processes in the vicinity of Alba Mons.

Conversely, the lowlands have been primarily shaped by sedimentary resurfacing (*55*), including by mud volcanism during the Late Hesperian to Early Amazonian (*49*). Despite these differences, the HCl subdetection points are situated above a significant fracture swarm characterized by volcanic landforms indicative of close interaction with igneous processes such as magma reservoir and dike emplacement (*57*) or in the vicinity of topographic ridges also interpreted as potentially related to dike emplacement (*49*), underscoring the critical role of fracture porosity in facilitating HCl seepage.

The shallow subsurface is ice rich, as testified in Scandia Colles by Phoenix lander observations (*59*) and everywhere by the widespread occurrence of the ice-rich (*60*) layered-dependent mantle (LDM) and associated morphologic patterns (*49*, *60*), including an endless succession of small (<10 m in scale) periglacial polygons associated with rubble piles in various abundances (figs. S4, S6, and S7). HCl of volcanic origin may be trapped in permafrost (*3*); the shallow subsurface may therefore act as a buffer for HCl of volcanic origin before ice sublimation and HCl seepage to the atmosphere, in agreement with the finding that volcanic areas are significantly enriched in Cl compared to the average Martian crust (*2*).

Because of uneven high-resolution imagery coverage, the geological sites beneath ACS HCl detections were explored to identify landforms meeting the criteria for potential HCl seepage sources (see Materials and Methods), not to find the seepage sites themselves. HiRISE and CaSSIS reveal possible seepage structures including fissures a few tens of centimeters wide, sometimes forming swarms up to tens of kilometers long on topographic ridges up to 30 m high and, in some cases, surrounded by dark haloes. They affect the LDM, dated 0.630 Ma (*61*), and their narrowness suggests that if not maintained until now, then they should disappear fast from the surface due to dust accumulation and seasonal surface deformation generated by isotropic periglacial activity. These structures have not been identified on Mars in earlier works. For instance, systematic geomorphologic mapping of the northern hemisphere did not consider these landforms (*62*–*65*). They align with large-scale tectonic and volcanic patterns (Fig. 2, A to D).

**Fig. 2.**
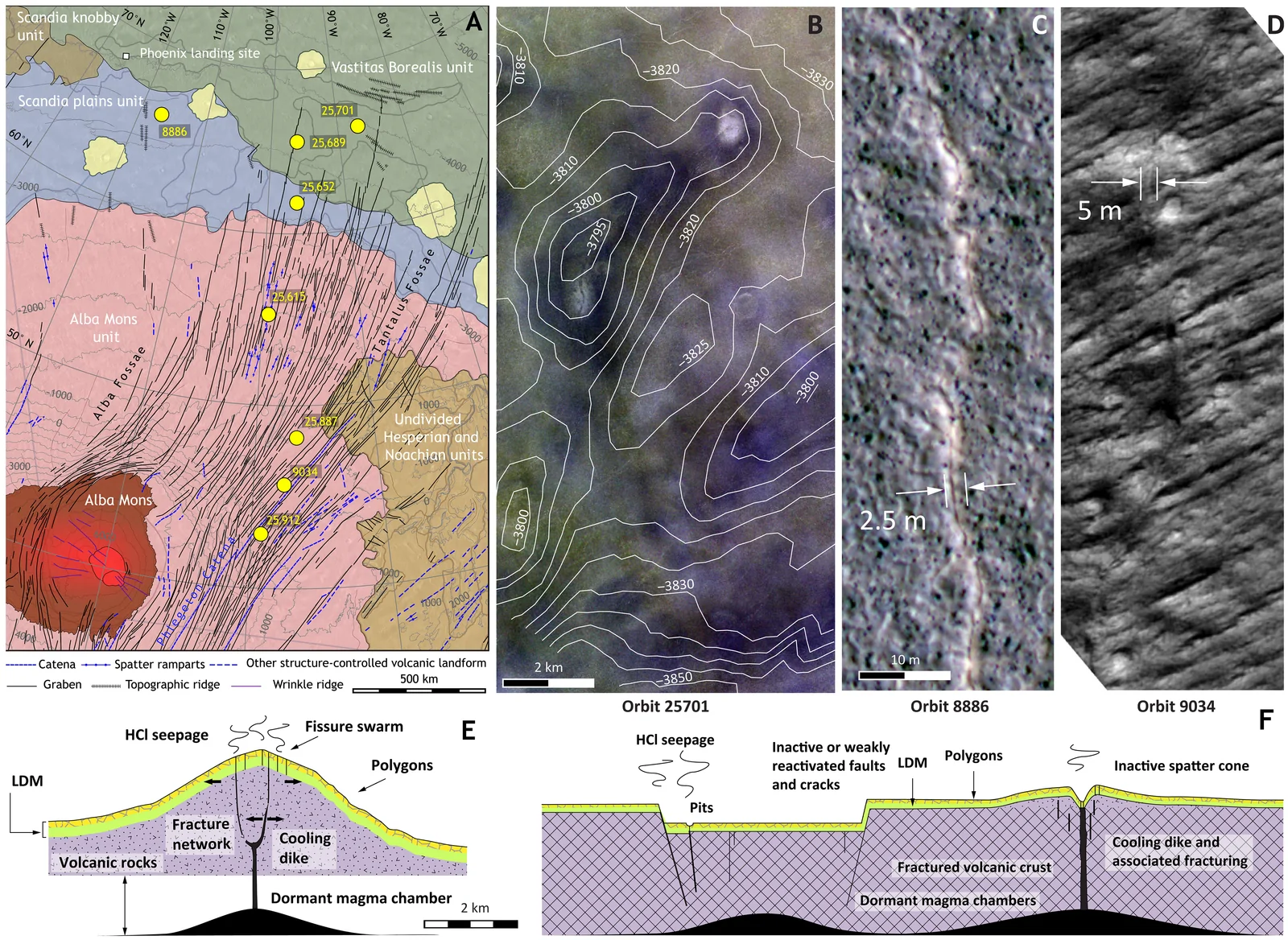
Geological context of HCl detection and possible seepage mechanisms. (**A**) Tectonic and volcanic map of the Alba Mons–Scandia Colles region. The base of the Alba Mons edifice is taken at an elevation of 4400 m. The lowermost HCl detections are located and named after their orbit number. They provide the central point of investigated geological sites. (**B**) Full-resolution CaSSIS image (MY37_023188_115_0, 4.6 m pixel^−1^) of a linearly fractured ridge (search site: orbit 25701). The ridge has bright and dark haloes suggesting local deposition. Topography is inferred from the MOLA tracks (see Materials and Methods). (**C**) Full-resolution HiRISE image (PSP_001431_2460, 25 cm pixel^−1^) of a fissure on top of a ridge in Scandia Colles (search site: orbit 8886). (**D**) HiRISE image (ESP_082648_2260, 50 cm pixel^−1^) of fissure in a graben (search site: orbit 9034). More examples are given in sections S3 to S8. (**E**) Geologic model for fissured topographic ridge formation in response to dike emplacement (lowlands). The extensional stress (horizontal arrows) in the LDM and polygonal terrain is controlled by dike emplacement. (**F**) Geologic model for Tantalus Fossae areas (highlands). The stress field is nondeviatoric or negligibly deviatoric. Vertical strain is not constrained.

Analysis of each HCl subdetection area (figs. S4 to S9) argues for a coherent crustal seepage mechanism, including outgassing from igneous activity at depth and interaction with the ice-rich subsurface. This two-level fracture system does not require any substantial thermal signature. The fractures may have initiated before resurfacing of the northern lowlands (*55*) in the Alba Mons geologic environment, propagating through the overlying sediments to the icy soil where tectonic stress dictated fissure geometry (Fig. 2, E and F, and figs. S4 to S9). The deep fractures would represent the top of a hydrothermal system connected to a volcanic plumbing system underneath, controlling a weakly deviatoric extensional stress field in which dike emplacement generates a ridged topography (*66*). In the case where fracture porosity is high because of igneous and tectonic interactions (Fig. 2F and figs. S6 to S8) (*57*), porosity would guide seepage with limited tectonic reactivation. Meter-scale pits aligned along narrow fissures or above subsurface cracks in the LDM would also form by ice sublimation exploiting fracture porosity.

The degassed HCl may be injected into the atmosphere in two possible ways: directly from the fractures or after interaction with the permafrost. Perchlorates fed by HCl of igneous origin would buffer interactions between HCl, permafrost, and seasonal atmospheric H_2_O (*67*). Perchlorate decomposition would generate new HCl molecules, which are those detected in the atmosphere. Moreover, leaching of basaltic fragments by subsurface liquids, if any, with rising HCl, might release molecular chlorine (*21*) that would recombine to HCl in the atmosphere.

## DISCUSSION

Localized aphelion detections of HCl at low altitudes, unexplained by observed atmospheric state and models, together with the geological context of the region, hint at an igneous origin of this gas. HCl was observed during the aphelion of MY 35 and MY 38 above the same area, suggesting a ground source that is persistent over an observational period, although it may be short lived on geological timescales. According to modeling (*42*) and observations (*4*), the uptake by ice particles is a particularly efficient atmospheric sink of HCl, yielding effective atmospheric lifetimes to be less than several sols for typical HCl abundances of a few parts per billion. Short lifetime is supported by our observations, as it prevents the gas from being mixed in the atmosphere and observed elsewhere during aphelion. Similarly, the same mechanism masks the source for observations throughout the year because of lower atmospheric temperatures and larger ice load.

Assuming that the observed aphelion HCl-rich air mass (∼1 ppbv) around Alba Mons represents less than 1% of the total Martian atmosphere (2.5 × 10^16^ kg) and adopting an model-effective atmospheric lifetime of 1 sol, we can estimate an upper limit on the seepage rate at <3 kg s^−1^. HCl fluxes from seeping sources at terrestrial volcanoes are commonly of the order of a few to >100 kg s^−1^ (*23*, *68*), and concentrations ranging from a few to ∼70 ppbv have been measured (*69*, *70*). While these terrestrial values cannot be directly extrapolated to Martian magmas or orbital detections, they provide an indication of the magnitudes that may be expected in the vicinity of an active source and are consistent with the derived estimate. For Mars, assumptions on passive degassing, noneruptive volcanic activity rates diverge by several orders of magnitude. Episodic degassing of water vapor from magma ascending to the shallow subsurface without erupting (*71*) was considered at rates 10^5^ to 10^7^ kg s^−1^ during events of largely unconstrained duration and recurrence. Applying similar release ratios to HCl yields emission rates several orders of magnitude higher than typical terrestrial volcanic outputs; these rates could only be reconciled with TGO observations if the events were extremely short lived. A much more conservative estimate, derived from scaling terrestrial volcanic degassing rates by 10^−3^ (*67*) on global and geological timescales, yields a mean HCl flux of ∼5 × 10^−3^ kg s^−1^ during the past 3 Ga (*72*). Such an estimate is more consistent with the dormant volcanic processes of unknown duration considered here.

During the warm perihelion season, observations yield a wide spread of HCl abundances of ∼3 to 6 ppbv at high latitudes in the Southern hemisphere. The ACS and NOMAD occultations (*28*, *33*, *34*) probe HCl at altitudes of 5 to 30 km, while astronomical observations (*73*, *74*) explore the lowermost altitudes to find similar abundances. Current three-dimensional climate-chemistry models invoke heterogeneous production as the sole source of HCl and generally reproduce the observations (*41*, *75*), but the rates of production and loss remain largely unconstrained, having been adjusted to reproduce TGO observations. The total mass budget of the HCl in the atmosphere during perihelion can be estimated as ∼3 × 10^7^ kg [assuming an HCl abundance of 5 ppbv at latitudes south of 30°S, corresponding to ∼25% of the total Martian atmosphere mass (2.5 × 10^16^ kg)]. Given that the models evaluate a median lifetime of HCl during perihelion of only a few sols (*75*), the insertion rate during this season is ∼180 kg s^−1^, nearly two orders of magnitude larger than an estimated volcanic seepage (<3 kg s^−1^). Thus, perihelion HCl is unlikely to be directly volcanic in origin. However, a connection between igneous and seasonal HCl may still exist via a mechanism complementary to heterogeneous production.

The overall cycle of hydrogen chloride includes irreversible sinks. In particular, global atmospheric transport simulations (*76*) demonstrate that chlorine from atmospheric HCl can be deposited to the surface and accumulate preferentially as perchlorates, in qualitative agreement with their widespread distribution. The atmospheric photochemistry models treat HCl uptake by ice as an ultimate sink as well, while its reverse release from frosts and clouds is envisaged (*73*, *74*). A portion of HCl may remain associated with water ice across the seasons and could be rereleased upon sublimation without requiring new heterogeneous production. Introducing a volcanic source of HCl, we can propose that a fraction of the perihelion HCl observed in the Martian atmosphere is ultimately of igneous origin. Localized igneous sources may therefore act as continuous primary contributors of chlorine, which is subsequently redistributed between atmospheric and surface reservoirs.

Notably, in contrast to other Martian volatiles that exhibit strong enrichment in heavier isotopes (most prominently water vapor, which is closely linked to the chlorine cycle), observed HCl shows no detectable isotopic fractionation of chlorine atoms (*38*, *77*). Several explanations are possible: (i) The chlorine isotopes observed in HCl did not participate in a long-term exchange cycle between the atmosphere and surface reservoirs; (ii) chlorine isotope fractionation mechanisms in the Martian atmosphere were inherently weak; (iii) because of its relatively high molecular mass and strong reactivity, HCl could not reach the upper atmosphere to escape; or (iv) the observed HCl has been introduced into the atmosphere relatively recently compared to the bulk of other volatiles. The current observational and modeling constraints are insufficient to distinguish among these scenarios. However, the hypothesis that the observed HCl isotopic ratio reflects a relatively recent injection into the atmosphere-connected reservoir remains compatible with the available observations.

The very young surface ages, of a few to tens of millions of years, reported for some Tharsis lavas (*5*, *6*, *8*), together with fault activity dated at >1 Ma (*7*), indicate that Tharsis remains seismically active. Cerberus Fossae provides further evidence for very recent or ongoing volcanism (*11*) and seismicity (*12*). Continued aphelion searches by TGO, particularly over the Tharsis and Cerberus regions, may therefore reveal additional localized HCl detections. Overall, the presence and variability of HCl in the Martian atmosphere appear to be governed by an interplay between episodic volcanic activity, atmospheric processing, and long-term sequestration into surface salts, perchlorates, and HCl-bearing ices.

## MATERIALS AND METHODS

### ACS data and processing

ACS is a set of three infrared spectrometers [near-infrared (NIR), MIR, and TIRVIM] onboard ExoMars TGO, aiming at atmospheric studies and, in particular, the remote detection of trace gases (*29*). ACS uses the solar occultation technique to provide information on Mars’ atmospheric vertical structure. The occultations are performed in an inertial pointing mode: For an ingress case, the line of sight extends from a tangent altitude of 250 km down to the point where it intersects the planet. The latitudes covered in solar occultation range from 88°N to 90°S and drift with date depending on orbital plane orientation. The best sensitivity to detect minor species is reached at 10 to 30 km, where the signal is not yet depleted by the aerosols, while the number of sounded molecules in the atmosphere is growing rapidly. Lowermost kilometers are not available because of attenuation of solar light on the line of sight at small tangent altitudes. The exact value of the lowest altitudes that can be reached by ACS during an occultation depends on the local aerosol load and varies from a few to a few tens of kilometers.

The cross-dispersion echelle ACS MIR spectrometer is dedicated to solar occultation measurements in the 2.2- to 4.4-μm range with high signal-to-noise ratio and fine spectral resolution. A steerable secondary grating allows one to choose between different groups of diffraction orders, changing the instantaneous spectral range. Typically, one occultation is dedicated to a certain secondary grating position. The current work is based on so-called positions 11 and 12, which both capture diffraction orders 173 to 175 (wave number range of 2894 to 3239 cm^−1^). This range contains HCl absorption lines, already used in ACS studies of hydrogen chloride (*4*, *28*, *34*, *35*, *38*, *43*). Retrieval of the HCl abundances is performed as described in (*38*).

Every ACS MIR occultation is also observed in parallel by the NIR channel. It is an acousto-optical tunable filter echelle spectrometer working in the 0.76- to 1.6-μm range, well suited to characterize CO_2_ and H_2_O profiles and provide information about aerosol continuum. The temperature and water vapor abundances are retrieved as described in (*37*). Temperature profiles are used to retrieve HCl from the simultaneous observations with ACS MIR.

The TIRVIM is a thermal-infrared channel with a 2-inch (5.08-cm) double pendulum Fourier transform spectrometer encompassing the spectral range of 1.7 to 17 μm with apodized resolution varying from 0.2 to 1.3 cm^−1^. ACS TIRVIM is primarily dedicated to profiling temperature from the surface up to ∼60 km and to monitor aerosol abundance in nadir (*78*, *79*). Derived thermal maps were used to search for warmth areas.

### Geological study of the ACS subdetection sites

The geological context of the ACS subdetection sites was inferred through the following steps:

1) We started with identification of the high-resolution datasets available at the ACS subdetection sites.

2) A distance of ∼1° of latitude was used around the lowest atmospheric detection, as a rule of thumb, to seek clues to potential seepage sources.

3) Potential seepage sources need to be specific to these geologic sites, as opposed to sources that would be potentially found at similar latitudes throughout the northern hemisphere. For instance, mud volcanism, periglacial polygons, or other landforms resulting from active permafrost processes may be present but are not, alone, criteria for potential seepage source identification. Pervasive surface blanketing by the LDM, which is ice rich (*60*), does not meet the specificity criterion either. On the contrary, association with local tectonic or volcanic features (*57*, *80*) meets the specificity criterion. In another example, periglacial polygons in Scandia Colles are associated with fissures that follow their geometry (*81*) (Fig. 2C) or the geometry of other periglacial landforms (*82*), indicating a related origin and therefore a possible widespread distribution in the northern hemisphere; however, alignment of these fissures with Alba Patera–related grabens or volcanic-tectonic depressions over kilometers or tens of kilometers in the studied sites (figs. S4 and S7 to S9) make them specific and satisfy the specificity criterion.

4) Landform dating is usually done using crater retention age calculation. This method cannot be applied here because seeping structures are expected to be much too small for crater statistics calculation to be relevant. Furthermore, definition of the boundaries of crater counting areas in the absence of a clear idea of what is to be sought is a major difficulty.

5) Landforms that are present on the most recent images but absent in older images are potential seepage sources. CTX imagery (resolution of 6 m pixel^−1^) makes this possible in many areas, due to multiple coverage of many areas, although for features of dimension larger than ∼15 m only. Comparison between CTX and CaSSIS images (resolution of 4.8 m pixel^−1^) locally allows to extend the time span. However, no such surface change could be identified using this approach. HiRISE stereoscopic coverage would also help follow potential surface changes but is not available in the search areas.

6) The Phoenix lander and heat shield degraded very fast on high-resolution (HiRISE) orbital imagery, with very significant changes apparent after one Martian year only. They cannot be distinguished from the surrounding boulders anymore. This suggests that at such high latitudes, meter-scale landforms that are not rejuvenated by seasonal processes, such as freezing/thawing cycles, may not be detectable anymore at these latitudes from orbit. As a consequence, we based our search of recent features on HiRISE images (*83*), with a resolution of 0.25 to 1 m pixel^−1^. When potentially relevant landforms were identified at this scale, such as the dark fissures identified at the highest latitudes, their appearance at lower resolution on HiRISE images with a resolution of 50 cm or 1 m, on color CaSSIS images (*84*), and, ultimately, on panchromatic CTX images (*85*), was used to diagnose them in regions where HiRISE images with a resolution of 0.25 m pixel^−1^ are not available.

7) Topographic information was sought to investigate the morphology of landforms of interest. Topography was retrieved using JMARS (*86*) along profiles with a vertical precision up to 40 m using the MOLA tracks (*87*). CaSSIS stereo pairs (*88*) were not taken in any of the sites of interest at the time of manuscript preparation.

### Geological mapping and model construction

The geologic map presented in Fig. 2A was prepared by coherently synthesizing the geological boundaries proposed in the two maps presented in (*49*, *89*). The unit names were taken from these maps. Topography is based on the HRSC-MOLA Blended Digital Terrain Model at a resolution of 200 m pixel^−1^ (*90*). Identification of volcanic and tectonic landforms reported on the map was done using CTX and HiRISE imagery (*83*, *85*). Dike and fracturing geometry at depth in Fig. 2E is inspired from experimental results of dike emplacement in which the initial stress field is close to isotropic and no tectonic stress is applied during emplacement. Stress is from gravity and the fluid pressure (*91*).

## Supplementary Materials

### This PDF file includes:

Supplementary Text Figs. S1 to S9 Table S1 References
