# Supplementary material for: Enigmatic HCl detections as a potential signature of igneous activity on Mars
Source: Sci Adv. 2026 Nov 4;12(45):eaeb9422. doi: 10.1126/sciadv.aeb9422 (PMC13733797; doi:10.1126/sciadv.aeb9422)
Supplement: Supplementary file 1 [file sciadv.aeb9422_sm.pdf]

Science Advances
AAAS

Supplementary Materials for

Enigmatic HCl detections as a potential signature of igneous activity on Mars

Alexander Trokhimovskiy *et al.*

Corresponding author: Alexander Trokhimovskiy, trokh@cosmos.ru

Sci. Adv. **12**, eaeb9422 (2026)
DOI: 10.1126/sciadv.aeb9422

This PDF file includes:

Supplementary Text
Figs. S1 to S9
Table S1
References

Supplementary Text

1. <u>HCl observations by ACS onboard TGO</u>

This dataset encompasses Trace Gas Orbiter (TGO) observations of Mars' atmosphere spanning from mid-Martian year 34 (Earth-year 2018) until the conclusion of Martian year 37 (2024). The occultation technique utilized by ACS enables the study of the vertical composition of the atmosphere. These measurements are sensitive to the aerosol loading, impeding observations at lower altitudes due to drastic light attenuation along the line of sight (*29*). The presence of HCl was revealed in a large number of occultations at high latitudes in both hemispheres and at all longitudes by ACS and NOMAD spectrometers during the perihelion period (*28*). Fig. S1A shows the distribution of HCl perihelion observations during MY 34-37, highlighting their regular seasonal occurrence. The corresponding areocentric solar longitudes (Ls) for perihelion detections range between 230° and 330°. The lower bound of the retrieved profiles during the dusty perihelion conditions varied between ~5 and 20 km depending on the dust load and latitude; the lowest altitudes were reached in relatively clean polar regions. High latitudes are also better sampled by TGO occultations. The derived profiles between solar longitude (Ls) 230° and 330° have abundances of 0.2–4 ppbv at altitudes ranging from 5 to 60 km. Abundances ≤ 1 ppbv are typical in the northern hemisphere, while higher abundances are found in the south. Notably, the southern-hemisphere HCl is vertically more spread with large values sometimes (Ls = 300–320°) observed down to the lowest altitudes probed, whereas the northern detections show a prominent maximum at 15–35 km (*34*, *35*). Outside the perihelion season, the HCl abundances drop below ACS detection capabilities ≤ 0.1–0.2 ppbv (except for cases considered in this paper).

In MY 37, during a dedicated campaign, six HCl detections were obtained over the same region and within the same Ls range as in MY 35 (*34*), also concentrating above the Alba Mons region (Fig. 1A, fig. S1). Similarly, to MY 35, approximately 1%-deep HCl absorption features were observed in spectra at several lowest transmittances, corresponding to low tangent altitudes. In the Alba Mons region, both detection and non-detection observations were obtained (fig. S1B and S1D). In MY 37, the first four detections (No. 3-6) are grouped at Ls=107°–111° at high latitude (55°-66°N) (see Table S1). The following detections (No. 7-8 were made shortly after Ls=117°–119° at significantly lower latitude (43°–48°N). All eight detections from two Martian years fall into a narrow longitude range -96° to -117°. During the MY 36 aphelion the latitude coverage of solar occultations did not include the Alba Mons region; also, in 2021 this season coincided with the solar conjunction when Mars was behind the Sun, and no science operations were possible between Ls=107° and Ls=113°. As a consequence, in MY 36 HCl could not be searched for in the period and location it was detected in MY 35 and further in MY 37.

The retrieved aphelion HCl profiles are presented in Fig. 1B shown in red, while profiles from the perihelion season are shown in blue. During aphelion, hydrogen chloride is restricted to the lowermost atmospheric layers, with a very steep gradient in the first 10 km. The maximum abundances measured in this season reach 1.5 ppbv. The measurements around the detection spots at latitudes 50–67°N are also close in time (fig. S1B and S1D), but provide only upper limits of ≤0.2 ppbv down to 2 km altitude (*34*). Similar upper limits are obtained at other longitudes all over the planet.

All aphelion HCl detections, in MY 35 and 37, as well as surrounding non-detection measurements in the northern hemisphere, were performed during Sun ingress (evening terminator). The local time of observations was from 19h to 3h (Table S1). No morning-terminators measurements were performed in this region.

2. Search for morphologic types corresponding to HCl seepage sources

Unequivocal identification of the seeping sites is out of reach with the available data; instead, we focus here on morphologic types in the vicinity of the ACS sub-detection sites which meet criteria of possible seepage-sources.

Assuming that seepage occurs within 1 degree of latitude of the geographical coordinates of the lowermost ACS HCl detection, the geology of each ACS sup-detection site is analyzed. Potential seepage sites must fill the following criteria:
1. Control by tectonic structures, plausibly connected to Alba Mons volcanism
2. Clue to very recent formation. In particular, the speed of degradation of Phoenix lander on HiRISE images (*83*) suggests that one Martian year is enough for ~1 m scale-landforms which are not seasonally reactivated by periglacial processes to become undetected from orbit after. Surface patterns which postdate the formation of the Latitude-Dependent Mantle (LDM), whose formation is controlled by obliquity variations and potentially as young as < 1 Ma (*61*) are also considered to be possible seepage sources, though at a lesser confidence level.

In the northernmost latitudes (TGO orbit numbers 8886, 25701), a common feature is the existence of swarms of fissures of width a few tens of centimeters arranged in segments extending over tens of kilometers, located on top of linear topographic ridges up to a few tens of meters high (figs. S4-5). Such linear fissures, showing a linear central trough bounded by bright exposures, have not been reported before, though they are morphologically similar to sinuous depressions at periglacial polygon scale (~a few meters) formed by permafrost processes described at a potential Phoenix landing site (*81*). At lower latitudes, similar fissures are observed both inside and outside Tantalus Fossae grabens, frequently parallel or perpendicular to graben trends (orbits 25689, 25652, 25615). Fissures are occasionally observed within flat-floored troughs of periglacial origin, and even guide their alignment, emphasizing interactions with permafrost (figs. S6-7). They are also observed on the LDM deposits within a spatter cone central vent, outside the spatter cone, and on impact ejecta which partly flowed onto the spatter cone central vent (orbit 25615). These fissures are significantly parallel to the spatter cone trend, suggesting reactivation of spatter cone-related fracturing (fig. S8). At the lowermost latitudes (orbits 9034, 25887, 25912), subsurface cracks guide sublimation pits located along Phlegethon Catena.

3. Orbit 8886 (MY 35, Ls=109.45, fig. S4)

Lowermost HCl detection coordinates: 65.8484°N, 116.853°W

Height of ACS detection above ground: 5400 m

Analyzed datasets (fig. S4A):

- CTX: Image mosaic (85) (6 m/pixel)

U01_071519_2328_XN_52N114W, F01_036348_2445_XN_64N119W, J02_045434_2446_XI_64N120W, P17_007655_2471_XN_67N119W, F01_036282_2445_XN_64N117W, F03_036849_2423_XI_62N115W, D20_035227_2448_XN_64N116W, J01_045302_2481_XN_68N118W, P17_007734_2489_XI_68N118W, N01_062906_2458_XN_65N116W, P18_007945_2488_XI_68N119W, J01_045091_2485_XN_68N119W, P18_008090_2461_XI_66N118W, F23_044880_2435_XN_63N118W, P01_001431_2459_XI_65N119W

- HiRISE images (83)
  PSP_001431_2460, ESP_045434_2445, ESP_046647_2475, PSP_001774_2460, ESP_026946_2465 (25 cm/pixel), ESP_027223_2455 (50 cm/pixel)
- MOLA altimetry measurements (*87*)
- HRSC MOLA blended digital elevation model (DEM) (*90*) 200 m

Geology

Overview

Exposed geological formation: Scandia region unit of Late Hesperian-Early Amazonian or Late Amazonian (*55*); Scandia Plain unit (*49*). The area is fully covered by LDM, which displays polygonal terrain and boulders, impact craters, and circular mounds interpreted as possible cinder cones or mud volcanoes (*49*). Similar polygonal terrain was also described in Phoenix landing site preliminary studies (*82*, *83*).

Unique features possibly associated with HCl seepage

- Linear topographic ridges with fissure swarms on top: Linear ridges are the only geomorphological features identified in the search area which meet the two criteria for potential HCl seepage sites.

One ridge, R1, was studied in particular (fig. S4D-E). MOLA and HRSC MOLA DEM indicate that this ridge is up to 10 m high. The top of the ridge shows narrow linear patterns parallel to the ridge trend. HiRISE image PSP_001431_2460 shows that these patterns include two bright stripes in the middle of which dark-toned lines of maximum thickness ~0.5 m are observed. Similar dark-toned lines, though guided by the geometry of periglacial polygons, were interpreted at a potential Phoenix landing site as depressions (*81*), perhaps resulting from thermal contraction (*92*). Such patterns are interpreted as fissure swarms of geometry probably related to topographic uplift in response to dyke intrusion (*49*), with deviatoric stress of tectonic and/or volcanic origin in addition to the horizontally isotropic thermal stress. The dark-toned depressions are continuous over tens to hundreds of meters, suggesting that they may be active, and are interpreted to be the result of interaction between deep tectonic, volcanic, and hydrothermal processes with soil movements generated by permafrost activity in the shallow subsurface.

- Fissures and fissure swarms: The other HiRISE images located on fig. S4A west of the orbit 8886 ACS observation show similar fissures, either following structural trend like at ridge R1 (e.g., ESP_045434_2445), or more randomly oriented (e.g., PSP_001774_2460, ESP_026946_2465), suggesting that they are widespread in the area, though with variable tectonic imprint. Those having clear tectonic imprint are those interpreted to be potential HCl

seepage sites, of which the fissure swarm at ridge R1 is the most spectacular. Their age is inferred from LDM to be several m.y. or less (*61*). East of this ACS observation, similar linear fissures are observed on image ESP_027223_2455. They are more scattered and not located on a topographic ridge; shades on the HiRISE image, confirmed by observations on CTX images, however, suggest that individual fissure segments may be located on individual ridges (e.g., 64°51'09"N, 115°02'18"W; 64°51'04"N, 114°54'49"W) which cannot be characterized using the available topographic information.

Summary
The identified fissures show evidence of ongoing activity. Such fissures in the search area (potentially not imaged by HiRISE) might correspond to HCl seepage sites.

4. <u>Orbit 25701 (MY 37, Ls=110.77, fig. S5)</u>

Lowermost HCl detection coordinates: 66.1735°N, 86.9356°W

Height of ACS detection above ground: 4600 m

Analyzed datasets (fig. S5A):
- CTX images (*85*) (6 m/pixel)
  F01_036149_2456_XN_65N087W, P01_001575_2467_XI_66N087W, D22_035938_2486_XN_68N088W, D22_035938_2486_XN_68N088W, F23_044681_2461_XN_66N086W, K21_061705_2461_XN_66N086W, F04_037217_2458_XN_65N085W, F03_036927_2456_XN_65N087W, G23_027235_2449_XN_64N084W, G03_019231_2430_XN_63N085W
- CaSSIS images (*84*) (4.8 m/pixel)
  MY37_023188_115_0, MY35_013548_110_0, MY36_022031_116_0
- HiRISE images (*83*)
  PSP_001575_2465 (25 cm/pixel), ESP_027235_2455 (50 cm/pixel), ESP_076700_2445 (50 cm/pixel)
- MOLA altimetry measurements (*87*)
- HRSC MOLA blended digital elevation model (DEM) (*90*) 200 m

Some of the CaSSIS and HiRISE images are beyond the 1° latitude of seepage source investigations, but show similar morphology at CTX scale and were investigated too.

Geology

Overview
Exposed geological formation: Vastitas Borealis interior unit, of Late Hesperian-Early Amazonian age (*55*); Vastitas Borealis unit (*49*). The area is fully covered by LDM, which displays polygonal terrain and boulders, impact craters, and circular mounds interpreted as possible cinder cones or mud volcanoes (*49*).

Unique features possibly associated with HCl seepage
- Fissure swarms culminating at bright patches. CaSSIS images of similar terrain located 1.5°lat and 3°lat south of the ACS detection reveal the presence of networks of fissure swarms connecting bright patches tens of meters high. The patches and occasionally, the swarms, are

surrounded by dark-tone haloes. In contrary to cinder cones and mud volcanoes, the bright spots are usually not circular. Their shape is irregular and they are occasionally elongated along the fissure swarms. We identified this fissure-bright patch association as potential volcanically-driven hydrothermal system, with seepage occurring either along patches, fissures, or both (fig. S5B-C). The dark halos may consist of redeposited dark seeping material. CTX images suggest that similar features are widespread over a broad area that encompasses the sub-HCl detections by ACS at orbit 25701.

Summary
Topographic ridges up to a few tens of meters high displaying fissures along strike, bright deposits on top, and dark flanks, are potential seepage sites. Furthermore, these patterns dominate the morphology, which reinforces the interpretation that such features are seepage sites.

5. Orbit 25689 (MY 37, Ls=110.32, fig. S6)

Lowermost HCl detection coordinates: 96.1999° W, 65.5545° N

Height of ACS detection above ground: 3400 m

Analyzed datasets (fig. S6A):
- CTX images (*85*) (6 m/pixel)
  F03_037046_2475_XN_67N097W, F22_044444_2458_XN_65N097W, D01_027486_2475_XN_67N096W
- CaSSIS images (*84*) (4.8 m/pixel)
  MY35_013511_110_0, MY35_007639_072_0
- HiRISE images (*83*)
  ESP_046633_2455, ESP_055428_2460 (50 cm/pixel)
- MOLA altimetry measurements (*87*)
- HRSC MOLA blended digital elevation model (DEM) (*90*) 200 m

Geology
Overview
Exposed geological formation: Vastitas Borealis interior unit, of Late Hesperian-Early Amazonian age (*55*); Vastitas Borealis unit (*49*). The area is fully covered by LDM, which displays polygonal terrain and boulders, impact craters, and circular mounds interpreted as possible cinder cones or mud volcanoes (*49*).

Unique features possibly associated with HCl seepage
- Fissure swarms. Fissures similar to those observed at MY 35 Orbit 8886 site are observed, though the highest resolution images are only 0.5 m pixel, preventing from good viewing of central dark depressions inside the two bright stripes (fig. S6Ba). The dominant fissure trend is parallel to the local Tantalus Fossae trend. The observations made here highlight the interaction between deep and surface processes:

- Fissure concentration is particularly large on the graben floor (fig. S6Bb), where the traces of the main normal faults are expected to be located. This suggests, though not demonstrates, that these faults may be preferential seepage sources.

- The cone slopes are affected by fissure swarms parallel to the main fissure trend, suggesting that either cone localization was influenced by deeply-rooted discontinuities related to the observed fissures (fig. S6Bc).
- A correlation exists between scalloped trough, of likely periglacial origin (*92*), and flat-floored depressions of probable similar origin, and the localization of the fissures outside the Tantalus Fossae graben, in particular west of the graben shown in fig. S6Bd. This suggests that fissure-related processes, in particular thermal, may help initiate the scalloped troughs, contribute to the release of potential HCl trapped in an ice-rich (*60*, *92*) LDM. The absence of scalloped troughs and flat-floored depressions at other HCl detection sites shows, however, that HCL release from LDM ice by scalloped trough formation alone cannot explain all the HCl aphelion detections.
- Local topography, in particular trough topography, significantly influences the localization and orientation of the fissures (fig. S6Be). Polygonal cracking results from horizontally isotropic stress, preferential localization and orientation denote the contribution of deviatoric body stress to fissure initiation and development.

Summary
Altogether, the observations made here show that although tectonics and igneous activity are important factor for HCl release, the fissures observed at the surface are probably superficial, perhaps restricted to the LDM, and rooted into deeper fractures, as interpreted in Fig. 2.

6. <u>Orbit 25652 (MY 37, Ls=108.94, fig. S7)</u>

Lowermost HCl detection coordinates: 96.1999° W, 65.5545° N

Height of ACS detection above ground: 3400 m

Analyzed datasets (fig. S7A):
- CTX images (*85*) (6 m/pixel)
  D01_027407_2421_XN_62N098W, F01_036334_2408_XN_60N095W, F23_044655_2410_XN_61N095W, J01_045354_2437_XN_63N096W, J01_045077_2422_XN_62N095W, J02_045420_2433_XI_63N097W
- CaSSIS images (*84*) (4.8 m/pixel)
  MY36_015621_068_0, MY36_022019_118_0, MY37_023035_068_0
- HiRISE image (*83*)
  ESP_019627_2425 (25 cm/pixel)
- HRSC MOLA blended digital elevation model (DEM) (*90*) 200 m

Geology
Overview
Exposed geological formation: Scandia region unit of Late Hesperian-Early Amazonian and Late Amazonian age (*55*); Scandia plains unit (*49*). The area is fully covered by LDM, which displays polygonal terrain and boulders, impact craters, and circular mounds interpreted as possible cinder cones or mud volcanoes (*49*). The area north of the ACS lowermost detection shows a boundary between two geologic units which have not been mapped earlier (fig. S7Ba). The terrain north of this boundary is lighter on THEMIS Day mosaic (*94*) and darker on CaSSIS images and displays a higher density of mounds or probable periglacial origin.

Unique features possibly associated with HCl seepage
- Fissures. Fissures similar to those observed at the MY 35 Orbit 8886 (fig. S4) site are also observed here at HiRISE scale, with maximum length 150 m (fig. S7Bb).
Fracture and fracture zones. Many fractures are observed on CaSSIS images, and shading, visible both on CaSSIS and HiRISE images, indicate that some are correspond to linear topographic highs (fig. S7Bc) or subtle linear slope variations. CaSSIS image resolution does not always permit to ascertain that the mapped lines are individual tectonic patterns, nor determine their kinematics, hence the fractures cannot be strictly named Fissures are in some cases better characterized as Fracture zones. In the CaSSIS-HiRISE overlapping area, the HiRISE image does not show that fractures zones are open at surface.

- Open fractures. On CaSSIS image MY37_023035_068_0, most fractures are dark and some display an inner linear bright pattern which might correspond to an open segment (fig. S7Bd). Their interaction with periglacial landform development is suggested by alignment of bright fracture segments with light-toned patches left by dark LDM erosion (fig. S7Be).

The overall fracture and fracture zone geometry shows two dominant orientations, parallel and perpendicular to Tantalus Fossae. The arcuate geometry of fracture patterns at proximity of the graben observed on CaSSIS image MY36_015621_068_0 suggests that some fractures are may be controlled by gravity; however, many fractures parallel to Tantalus Fossae are far away from grabens and are unlikely to be influenced by body forces. The fracturing patterns are interpreted as pervasive brittle deformation structures affecting at least the first hundreds of meters or kilometers of the crust.

Summary
The area is pervasively fractured, with local evidence of interactions between deep fractures and with permafrost processes. Open fissures on HiRISE images and open fractures on CaSSIS images, or similar structures not covered by the available HiRISE nor CaSSIS images, might be the locus of HCl seepage.

7. <u>Orbit 25615 (MY 37, Ls=107.56, fig. S8)</u>

Lowermost HCl detection coordinates: 99.3731° W, 55.2134° N

Height of ACS detection above ground: 2300 m

Analyzed datasets (fig. S8A):
- CTX images (*85*) (6 m/pixel)
  G02_018902_2317_XN_51N100W, D02_027882_2299_XN_49N101W, K21_061666_2369_XN_56N099W, U02_071558_2351_XN_55N099W, D02_027961_2380_XN_58N099W, D20_035121_2344_XN_54N099W D04_028660_2323_XN_52N100W, K01_053859_2324_XN_52N100W, J02_045552_2324_XN_52N099W P16_007127_2319_XN_51N097W, U18_077914_2346_XN_54N098W
- HiRISE image (*83*) (50 cm/pixel)
  ESP_025706_2360

- MOLA altimetry measurements (*87*)
- HRSC MOLA blended digital elevation model (DEM) (*90*) 200 m

Geology

Overview

The area was mapped (*55*) as part of the Alba Mons unit, or Late Hesperian to Early Amazonian age. The landscape is mantled by LDM affected by periglacial polygons.

Unique features possibly associated with HCl seepage

- Fissures. The area is intensely fractured by Tantalus Fossae grabens, and the sub-point of the lowermost orbit measurement is a few kilometers east of a long chain of spatter ramparts. The available HiRISE image of shows the inner part of the vent between the two ramparts (fig. S8B). Narrow fissures similar to those observed in the area of detection of e.g. Orbit 8886 (fig.S4D) are pervasive in all the mapped units. The length of many of the fissures, up to 236 m (fig. S8C), and low sinuosity (fig. S8D), indicates that their geometry was not controlled by periglacial processes related to polygon formation. Their orientation, parallel to the spatter ramparts (fig. S8C), suggests reactivation and upward propagation to the LDM of spatter rampart-related structures. Depending on the age of this LDM, the fissures may be only a few million years old or younger (*92*).

Summary

There is evidence that some volcano-tectonic structures along the spatter ramparts at proximity of detection of Orbit 25615 were reactivated very recently, giving birth to narrow fissures. Such features may potentially be related to HCL seepage above a dormant magma chamber.

8. <u>Orbits 25887, 9034, 25912 (fig. S9)</u>

- Orbit 25887 (MY 37, Ls=117.79)

Lowermost HCl detection coordinates: 96.5837° W, 48.1901° N
Height of ACS detection above ground: 2700 m

- Orbit 9034 (MY 35, Ls=115.01)

Lowermost HCl detection coordinates: 97° 27' 00" W, 45° 42' 36" N
Height of ACS detection above ground: 900 m

- Orbit 25912 (MY 37, Ls=118.75)

Lowermost HCl detection coordinates: 99° 16' 12.72" W, 42° 53' 43.44" N
Height of ACS detection above ground: 2474 m

Analyzed datasets (fig. S9A):

- CTX images (*85*) (6 m/pixel)
  B19_016924_2255_XN_45N096W, B19_017201_2260_XN_46N099W, D19_034699_2295_XN_49N096W, D20_035187_2238_XN_43N099W, D21_035332_2260_XN_46N098W, D22_035833_2289_XN_48N096W, F01_036321_2241_XN_44N097W, G01_018559_2275_XN_47N096W, G02_019126_2279_XN_47N095W, G20_025996_2285_XN_48N094W, G21_026418_2256_XN_45N095W, G21_026616_2232_XN_43N100W,

G23_027262_2243_XN_44N095W, G23_027328_2236_XN_43N097W, J01_044985_2238_XN_43N100W, J02_045697_2280_XN_48N097W, J03_045842_2287_XN_48N097W, J04_046264_2250_XN_45N097W, J04_046409_2246_XN_44N097W, J05_046620_2254_XN_45N098W, J07_047398_2262_XN_46N098W, J20_052514_2272_XN_47N097W, J21_053160_2284_XN_48N095W, K04_055059_2238_XN_43N099W, K15_059332_2220_XI_42N099W, K17_059833_2224_XI_42N098W, K20_061112_2267_XI_46N095W

- CaSSIS images (*84*) (4.8 m/pixel)
  - Orbit 25887: MY36_016135_132_0, MY35_007183_134_0, MY36_016048_133_0, MY36_015695_051_0, MY36_017013_048_0
  - Orbit 9034: MY35_011679_050, MY34_005517_132
  - Orbit 25912: MY36_015720_047, MY35_013462_135, MY36_016073_137, MY37_023301_137
- HiRISE images (*83*)
  - Orbit 25887: ESP_023939_2285, ESP 067891_2285, ESP_076727_2285, ESP_076384_2285 (25 cm/pixel), ESP_023517_2285 (50 cm/pixel)
  - Orbit 9034: ESP_050167_2260, ESP_019126_2270 (25 cm/pixel), ESP_047398_2265, ESP_046409_2255, ESP_082648_2260 (50 cm/pixel),
  - Orbit 25912: ESP_070055_2235 (25 cm/pixel), ESP_028238_2240 (50cm/pixel), ESP_035187_2240 (50 cm/pixel)
- HRSC MOLA blended digital elevation model (DEM) (*90*) 200 m

Geology

Overview

The area was mapped (*89*) as part of an Amazonian and Hesperian volcanic unit. The detections are around Phlegethon catena, the longest pit chain observed in the Alba Mons region. The pit chain includes pit craters as well as other depressions, possibly including diatremes. Pit craters were interpreted as surface manifestation of underlying dykes, and the whole catena as a volcano-tectonic feature located above elongated magma chambers from which the dykes intruded the overlying crust (*80*).

Many grabens were partly filled by the LDM, and depending on their topography with respect to these grabens, the LDM is observed to have flowed into some of the pits are therefore partly filled by the LDM as well.

Unique features possibly associated with HCl seepage

Linear troughs along pit and graben walls. When mantled by LDM, some of the grabens and pits of Phlegethon Catena display linear collapse troughs taking the form of small circular or elongated depressions commonly less than 50 m in width formed along an underlying crack or fault. These depressions are either observed along the walls of pits, therefore conforming to their shape in map view, or aligned along normal faults in grabens. In a few cases, the troughs are located in the central part of a graben, or are observed to have propagated both on wall slope and toward the central part of a graben, which indicates that body forces did not play a critical role in fissure propagation. As a consequence, the fissures are interpreted as a consequence of tectonic

reactivation of catena structures, whether graben faults or pit-related fractures, and collapse features are interpreted as sublimation pits controlled by these fissures.

An exhaustive survey of catenae throughout the Alba Mons region revealed that such collapse troughs are exclusively observed in Phlegethon Catena between the ACS sub-detection points of orbits 25887 and 25912. They are potential seepage sites for observations made at orbits 9034, 25887 and 25912 (fig. S9A). Fig. S9B-E illustrates reactivated structures and possible seepage sites.

Similar troughs are observed northeast of Alba Mons at ~46°N, 130°W. No ACS observations were made close enough to them to investigate a correlation with potential HCl seepage sites at that place.

Summary
Linear troughs formed in the LDM of catenae along subsurface cracks or faults found at Phlegethon Catena and remarkably, only in the areas of HCl detections made at orbits 9034, 25887 and 25912. They are therefore thought to have formed recently and are potential seepage sites. Trough formation may be related to preferential ice sublimation along the subsurface structures, explaining why they are not observed in e.g. the lava flows when exposed at the surface. The involved tectonic structures are interpreted to be reactivated graben structures (normal faults or tension cracks). It may be speculated that the reason why many fault scarps in this area are fresh-looking might be due to reactivation too.

9. Potential HCl sources and sinks in the atmosphere

Chlorine is a relatively abundant element on Mars, its content increasing from bulk values 32–39 ppmm (*95*) to 0.5 wt% at the 10–50 cm depth (*96-98*) and up to 1.28 wt% in the uppermost surface layers (*99*). Chlorine abundance inferred from GRS/ODY data is correlated with H abundance and anti-correlated with thermal inertia (*96*) suggesting higher Cl abundance in weathered surfaces compared to rocky surfaces. The surface dust is also enriched in Cl (*100*). Overall, Cl distribution is generally accepted to be controlled by aeolian deposition and surface alteration (*98*). Following the discovery of atmospheric HCl, its chlorine isotopic composition (*38*, *77*) was found to be consistent with meteoritic (*101*) and Martian soil values (*42*), supporting a link between HCl and chlorine-bearing aerosols. In contrast to other volatile species on Mars, HCl did not exhibit measurable isotopic fractionation, the measured $^{37}Cl/^{35}Cl$ ratio is almost indistinguishable from the terrestrial ratio for chlorine.
Four possible pathways of HCl production from dust were proposed (*28*) including heterogeneous oxidation of the mineral aerosol by water vapor dissociation products, such as OH and HO_2; oxidation by acids, which might hypothetically be present in the Mars' atmosphere; UV activation of surface dust freshly exposed by dust storms; or activation by electrostatic discharges during dust storms. Electrostatic discharge (ESD) activity in the form of lightning in dust storms or small-scale discharges in dust devils has not been directly observed on Mars but is expected and may be a source of Cl-bearing atmospheric compounds (*102*).
Observations of HCl during perihelion season show a noticeable hemispheric dichotomy in distribution (*28*). Southern-hemisphere abundances are rather uniform in the lower atmosphere and reach 5 ppbv, while the northern-hemisphere abundances are all below 2 ppbv and have a prominent maximum at altitudes of tens of kilometers (*34*). Equatorial observations also do not

reveal the presence of HCl at similar dates. Taken together, these observations suggest that the southern summer is the dominant source of HCl injection, from which trace gases, including hydrogen chloride and water vapor, are transported at high altitudes to the opposite hemisphere by the Hadley circulation (*43*). This flow of humid air descends in the Northern hemisphere winter, where water condenses, producing ice clouds that could trap HCl molecules (*4*, *42*), thereby preventing global mixing.

Although HCl was discovered in MY 34, characterized by a perihelion global dust storm, further observations demonstrated that the appearance of HCl does not necessarily require a dust storm, but rather coincides with the warmer, dustier, and more humid perihelion season. A pronounced correlation between HCl and water vapor (*33*, *43*) supports only the first of the mechanisms out of the mechanisms listed above, leading to a number of modelling efforts (*40*, *41*, *103*). In one of the models, the majority (96.5%) of gaseous chlorine production is driven by reactions between chlorides and molecular hydrogen (*40*). During perihelion, this pathway can be considered primarily at elevated altitudes, where the H_2O photolysis produces enough of odd hydrogen with lifetimes exceeding several seconds. Alternatively, HCl can be produced in reactions of Cl with HO_2 (*28*, *33*), the source of Cl being perchlorate decomposition within the dust grains (*42*). The highly reactive HO_2 is expected to have several-times-higher concentrations during perihelion (*104*) with peak abundances at ~30 km altitude, in qualitative agreement with the observed HCl profiles. The rapid decline of HCl after the end of the perihelion season can be explained by uptake of HCl on water ice grains and $CaCO_3$–bearing dust particles, reducing its lifetime from ≥90 sols to 1–10 sols in the 5–35 km altitude range (*33*, *42*).

The temperature affects the proposed perihelion heterogeneous HCl production through water vapor saturation and, hence, the availability of odd hydrogen and its products. It also controls cloud formation and the HCl uptake mechanism, a process known in the terrestrial atmosphere (*105*, *106*) and acknowledged to occur on Mars (*4*), through its negative dependence on temperature. In the Martian atmosphere, cloud particles form extensively at temperatures below ~200 K while the heterogeneous uptake rate of HCl(g) onto ice, derived from Earth stratospheric pressures and temperatures, falls sharply above 180 K (*107*, *108*). Below 180 K, HCl is adsorbed easily and removed quickly, while the warmer perihelion temperatures allow it to persist longer in the atmosphere.

Despite a number of difficulties and assumptions the models can reproduce the HCl abundances, the seasonal trend and some features of its perihelion distribution by balancing heterogeneous production and loss. However, the localized hydrogen chloride abundances, observed during the aphelion season are not consistent with the above mechanisms. The HCl mixing ratios obtained in two different seasons are of the same order of magnitude (ppbv), while summer in the Northern Hemisphere is roughly 30 K colder than in the southern hemisphere. As a consequence, neither potential mechanism is valid, the H and HO_2 abundances in the atmosphere are very limited compared to the perihelion season.

There are no indications of any perceptible longitudinal warming that could cause enhanced HCl production to the north of Alba Mons. We have examined temperature profiles measured by ACS NIR simultaneously with ACS MIR observations. The temperature profiles of aphelion detection orbits (fig. S2, panel A, red curves) do not differ from those accompanying many non-detections in the Northern hemisphere (black curves), suggesting that the thermal state of the atmosphere does not control the localized hydrogen chloride formation. Warmer profiles are measured at later dates of the investigated period, Ls range 115°–119°. The temperatures from warm perihelion southern summer, when HCl is widespread, are shown in blue for reference.

A thermal map of the Northern Hemisphere atmosphere as derived by ACS TIRVIM nadir measurements in MY 35 for the Ls range 110°–116° is presented at fig. S3 panels A and B. The right edge of the TIRVIM observations Ls range is limited by instrument data availability. The presented maps correspond to the 10 kilometers altitude. Panel a shows daytime temperatures, local time from 14 h to 22 h, panel b shows nighttime, local time from 22 h to 6 h. The observation points for ACS MIR are denoted by circles, with solid-filled circles indicating HCl detections. A daytime warming can be identified above elevated Tharsis and Alba Mons. However, the northern part of the Alba Mons region is only at the edge of the warm spot. And as observed by ACS NIR in occultations, HCl detections are observed along with temperatures that are not anomalous compared to those measured at other longitudes. Nighttime temperatures are uniform across longitudes. TIRVIM is also capable of retrieving surface temperatures, daytime and nighttime results are presented at fig. S3 panels C and D. Daytime surface temperatures at Alba Mons match those of other longitudes, whereas certain southern regions become cooler at night.

Since HCl typically demonstrates good correlation with water vapor abundances, we have also checked water vapor profiles simultaneously measured by ACS NIR (fig. S2C). Similarly, to temperature profiles, no particular differences were found between HCl detection orbits and other locations. The ice load is believed to rapidly remove HCl from the atmosphere, its content was measured by ACS TIRVIM (fig. S3E). Lower altitudes are characterized by a thick aphelion cloud belt, while higher altitudes are much cleaner, although measurable ice loads persist. The Alba Mons region exhibits slightly elevated values, implying that HCl uptake must be even more efficient than at other longitudes.

The HCl profiles we retrieved and their spatial distribution for these localized aphelion detections show that the gas is not well mixed in the atmosphere either vertically or horizontally. The source is located close to the surface at the locations of detections, in the near-surface atmosphere, at the surface itself, or in the subsurface. Overall, observations of the atmospheric state suggest that the source is non-atmospheric and is plausibly located within subsurface fractures.

10. Rationale for HCl vs SO_2 degassing

Localized aphelion detections of HCl at low altitudes, together with the geological context of the area, suggests igneous an origin of this gas and that the detections are made above the seepage areas. Another major volcanic gas exsolved from Martian magmas is SO_2 (*27*, *109-111*), and the abundance of sulfate deposits older than 3 Ga testifies to the release of large amounts of sulfur from the Martian interior and its storage in sediments during the construction of most of the large igneous provinces of Mars (*112*).

Having the SO_2 degassing estimate (*111*), and assuming the HCl/SO_2 ratio on Mars being twice of that for terrestrial arc volcanoes (*24*, *72*, *113*), a nominal case of Martian volcanic degassing with the SO_2 flux was constructed being on average 1.7 times stronger than that of HCl (10^5 against $6 \cdot 10^4$ molec cm^{-2} s^{-1} (*72*). In their atmospheric model, these rates result in 0.08 ppbv of SO_2 and 0.02 ppbv of HCl in the whole atmosphere. Attributing a fraction of Mars volcanic degassing to recycling of the crust volatiles, even a higher HCl/SO_2 degassing ratio of ~1 was inferred (*114*). Keeping the arbitrariness of the global estimates in mind, the HCl detections reported here (~1 ppbv) should correspond to 1–4 ppbv of SO_2 that is compatible with astronomical upper limits ranging from 0.3–1 ppbv over the planet's disc to 3 ppbv above

Tharsis and Syrtis Major regions (*17–18*). The lifetime of both gases in the atmosphere is ~1 MY (33, 116, 117) preventing preferential accumulation of either SO_2 or HCl.
In the ACS spectra, the sulfur gases were searched (*117*) during the MY 34 and 35 perihelion seasons to derive upper limits for SO_2, H_2S and OCS at 20, 15 and 0.4 ppbv, respectively. The SO_2 absorption in the ACS spectral range is weak and more stringent upper limits were established in the microwave (*19*) or longer IR (*17*, *18*) spectral ranges. The H_2S and OCS abundances are expected to be 50–200 times lower than those of SO_2 or HCl (*22*, *23*).
On Earth, degassing of SO_2 is known to depend on the geodynamical environment of the volcano; for instance, it has been rare or even absent in many arc volcanic eruptions in Kamchatka (*20*, *21*, *118*). It was also shown (*20*, *119-125*) that SO_2 emissions are inversely correlated with HCl emissions and particularly low during post-eruptive periods. Due to its higher solubility in magma compared with SO_2, HCl continues to be degassed after the end of volcanic activity (*126*), for instance, from lava flows (*119*), but also from the remaining magmas at depth, producing chlorine vent fields (*21*). Thermodynamic models have shown that chloride plumes are characteristic of volcanic emissions in cold climates rather than sulfate plumes (*127*), which is supported by experiments showing increased HCl adsorption by ash with temperature across the terrestrial and Martian temperature ranges (*128*). The subdued topography of many fractures and impact craters in the Alba Patera and Scandia Colles regions is consistent with substantial tephra deposits. The non-detection of sulfur compounds associated with chlorides in ACS observations may thus be explained by the cold Martian climate and the absence of ongoing eruptions. Interestingly, interactions between volcanic gas emissions during small, sporadic ash events and underlying hydrothermal systems may substantially increase the HCl/SO_2 ratio (*129*).
In summary, ACS observations of HCl without detectable sulfur emissions may reflect dormant volcanic activity in a cold environment, without precluding episodic, limited pyroclastic eruptions that leave little detectable trace on the surface in the absence of a landed mission.

№	Orbit number	Ls	MY	Latitude (tangent altitude 10 km)	Longitude (tangent altitude 10 km)	Sub-detection site surface elevation wrt areoid, km	Local time, hours
1	8886	109.45	35	65.83	-117.05	-3.7	23.23
2	9034	115.01	35	45.71	-97.45	-0.3	19.68
3	25615	107.56	37	55.45	-99.77	-1.4	3.3
4	25652	108.94	37	61.96	-96.69	-3.2	2.27
5	25689	110.32	37	65.59	-96.55	-3.6	1.03
6	25701	110.77	37	66.19	-87.25	-3.9	0.6
7	25887	117.79	37	48.31	-96.44	-0.4	19.8
8	25912	118.75	37	43.04	-99.12	0.2	19.47

Table S1.
List of ACS MIR aphelion HCl detections sites.

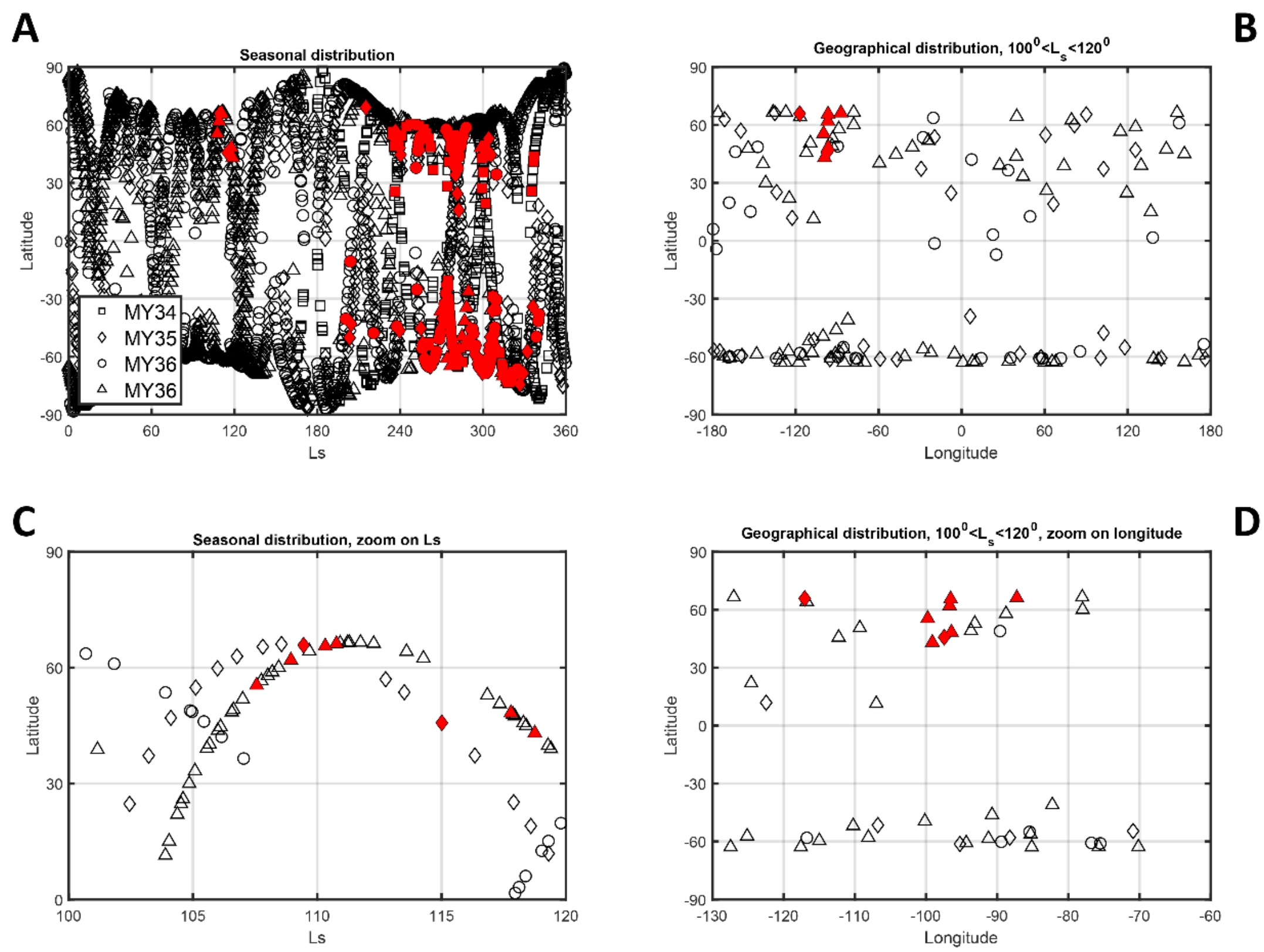

Fig. S1.

Seasonal and spatial distribution of HCl perihelion observations during MY 34-37. (A, C) Seasonal distribution of ACS MIR observations and HCl detections - in red. Different markers stand for different Martian years as specified in the legend. Lower panels C shows a zoom on aphelion detections. (B, D) Geographical distribution of ACS MIR observations and HCl detections - in red, the corresponding L_s = 100°-120°. Lower panel D shows a longitudinal zoom.

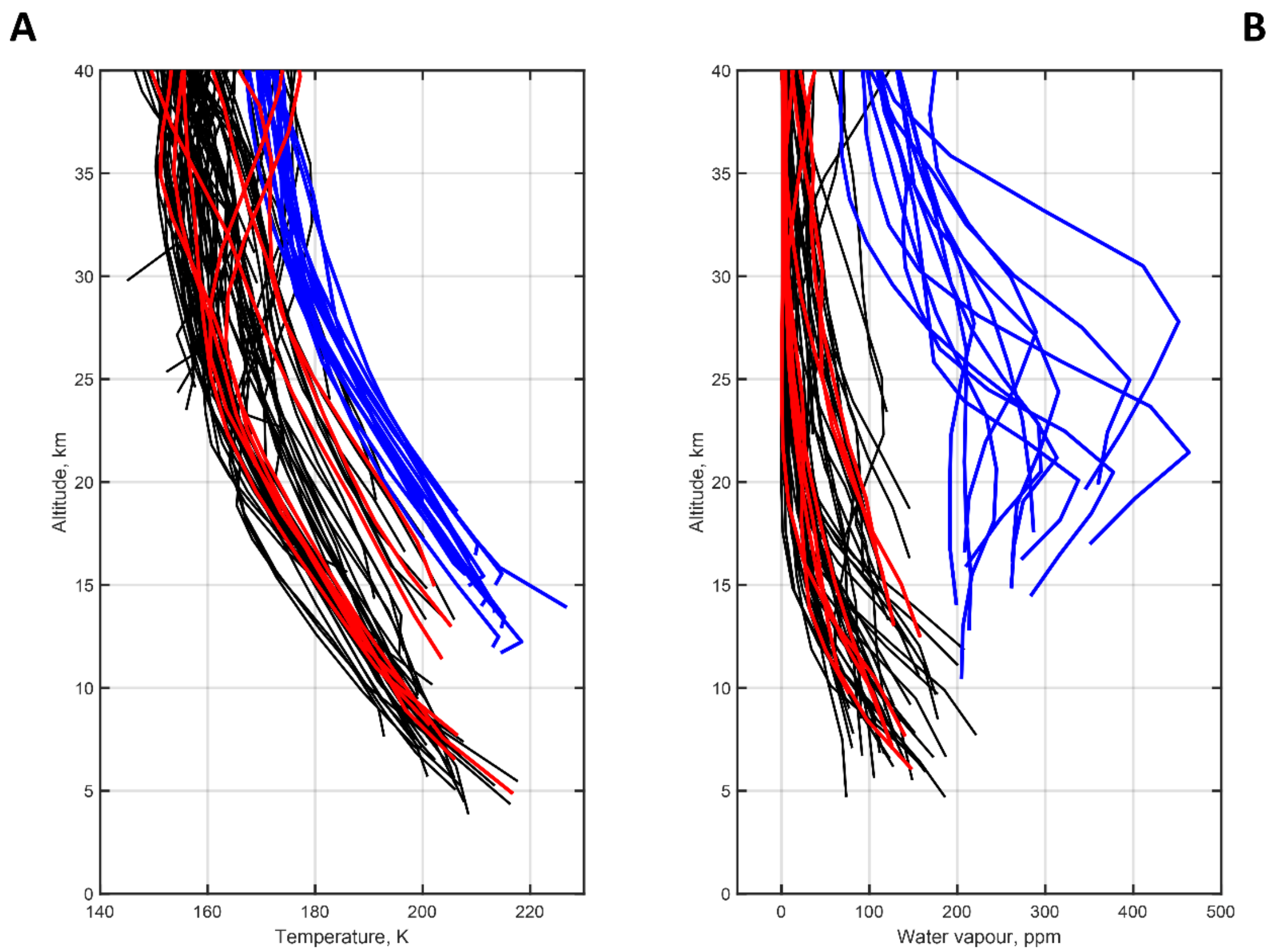

Fig. S2.

Atmospheric state as measured by ACS NIR during the occultations. (A) Temperature profiles. In red - northern hemisphere observations with HCl detections, in black - northern hemisphere observations without detections for L_s = 100°-120°, in blue - profiles at HCl rich perihelion season from Southern hemisphere for a comparison (latitudes (66°-53°S), L_s = 292°-304°, MY 35). (B) Water vapor profiles, sampling and notations as on panel .

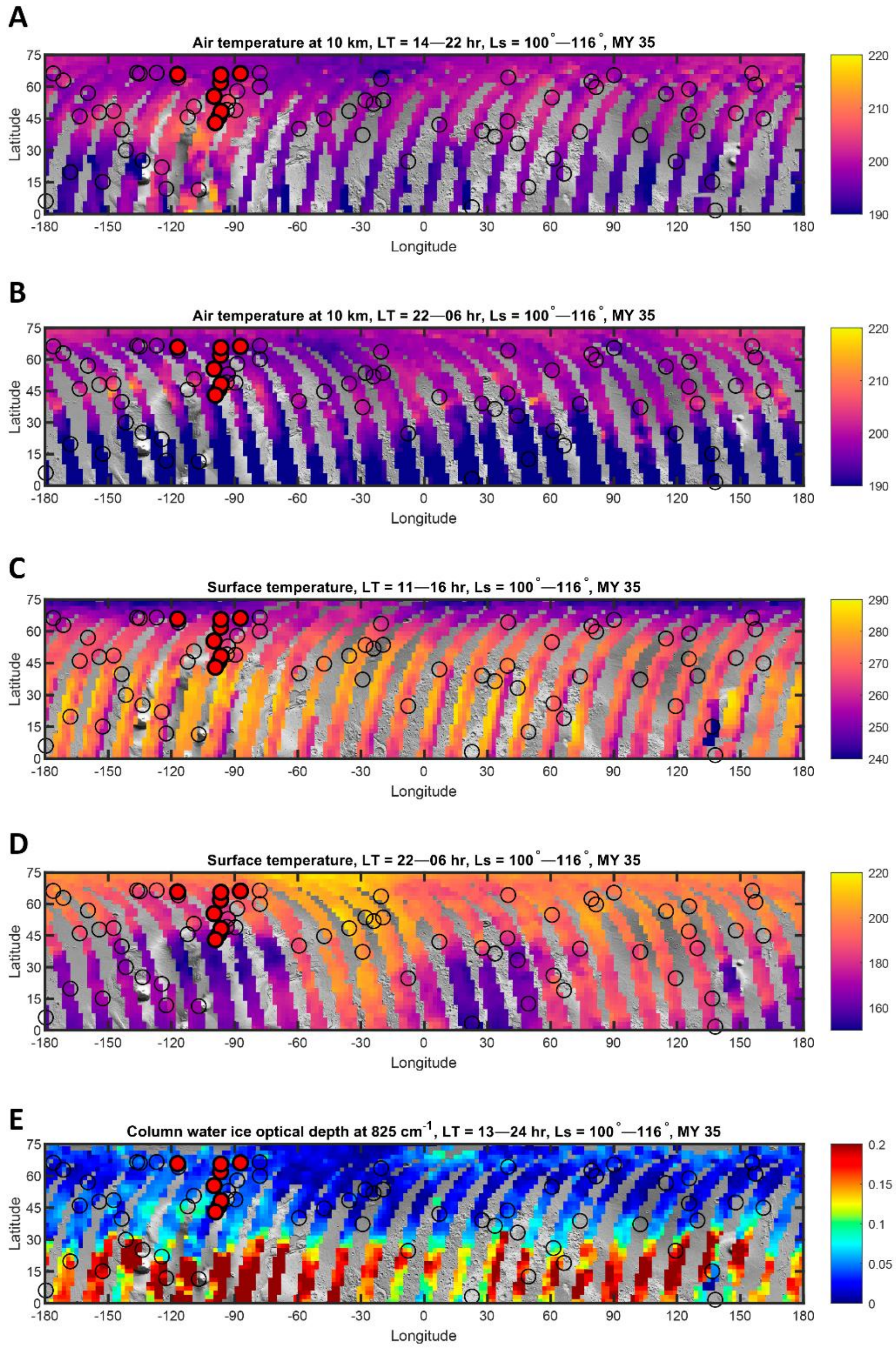

Fig. S3.

Thermal maps of the atmosphere and ice load as derived by ACS TIRVIM in MY 35 for the Ls range 110°-116°. (A) Daytime atmospheric temperatures at 10 kilometers elevation altitude. (B) Night time atmospheric temperatures at 10 kilometers elevation altitude. (C) Daytime surface temperatures. (D) Night time surface temperatures. (E) Column water ice optical depth at 825 cm^{-1}.

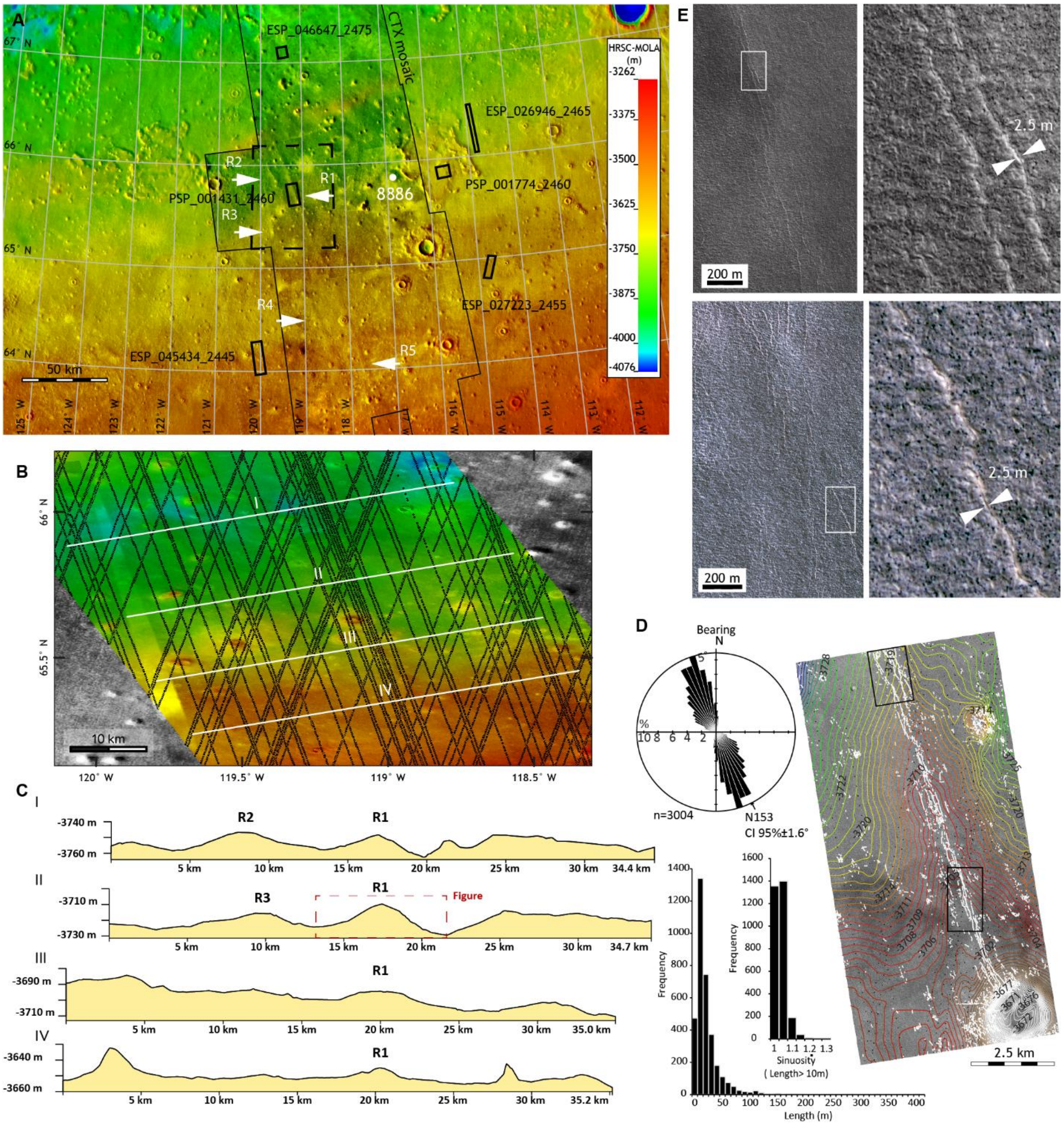

Fig. S4.

Interpretation of geology at Orbit 8886 ACS occultation. (4A) Location of topographic ridges (R1-5) in Scandia Colles interpreted to be of volcanic origin (arrows); the lowermost HCl detection by ACS (white dot); the analyzed CTX mosaic and HiRISE images; and location of (4B) (dashed box). The background map is the THEMIS Day image mosaic draped over the HRSC MOLA blended digital terrain model (DEM). (4B) Location of topographic profiles R1-3 displayed on (4C). Topography is inferred from the MOLA shots (black dots), and is very similar to the HRSC MOLA blended DEM topography. (4C) Topographic profiles across ridges. R1 is interpreted in (4D). The red box locates the topographic profile used in the article in Figure 2. (4D) Interpretation of HiRISE image PSP_001431_2460. White lines are fissures. Contours are

interpolated from the MOLA shots (black dots). The circular mound may be either volcanic, or a mud volcano formed by destabilization of subsurface volatiles by magmatic heat underneath. Sinuosity is given for fissure lengths > 10 m, below which the influence of polygon geometry becomes patent. The two black boxes locate the HiRISE zooms (4E) (top: PSP_001431_2460_RED; bottom: PSP_001431_2460_COLOR). In the Bearing plot, CI = Confidence interval; n = number of mapped fissures. (4e) HiRISE views of fissures on top of topographic ridge R1. Images PSP_001431_2460_RED (top) and PSP_001431_2460_COLOR (bottom).

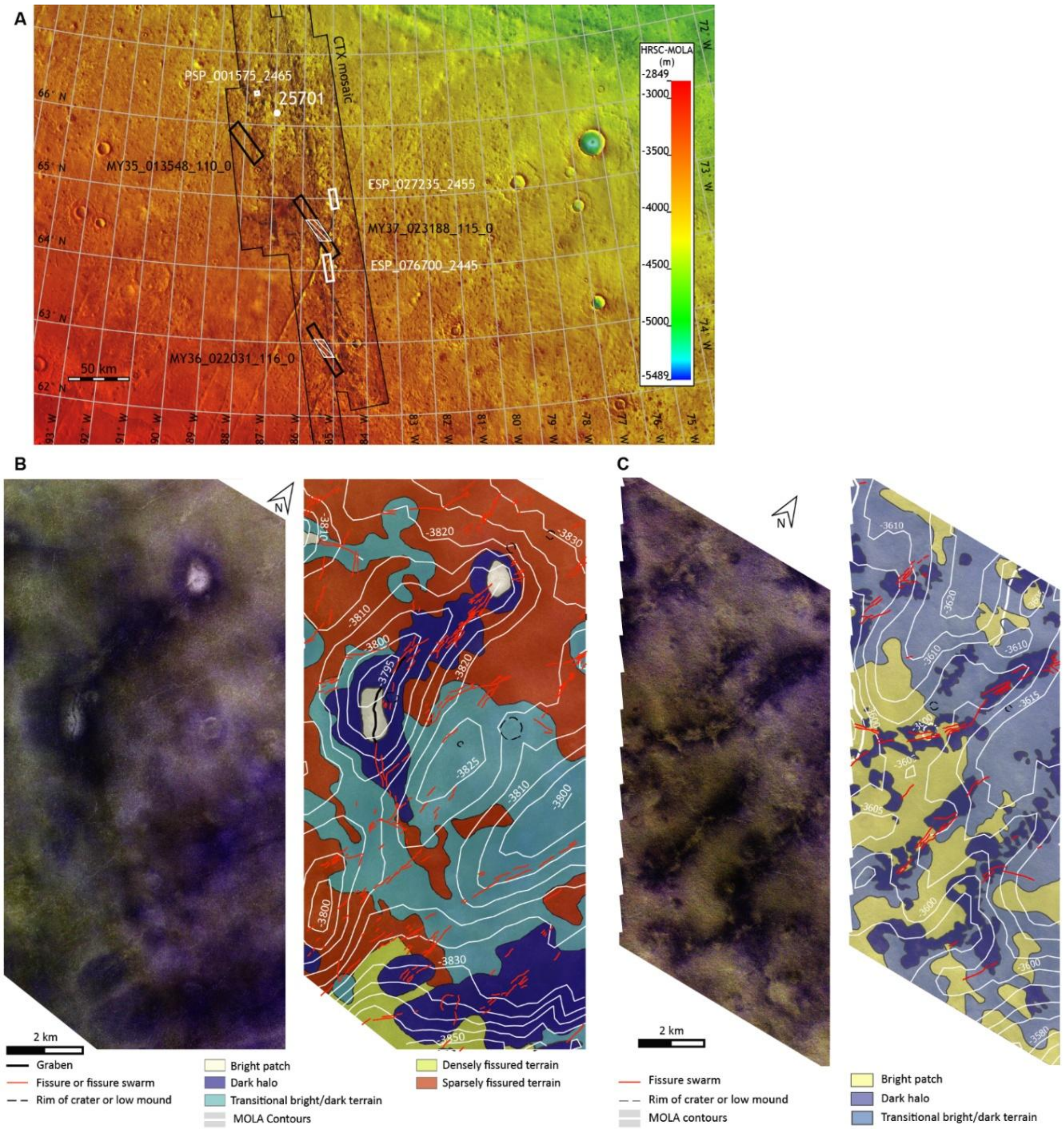

Fig. S5.

Interpretation of geology at Orbit 25701 ACS occultation. (5A) Location of the lowermost HCl detection by ACS (white dot), and the analyzed CaSSIS, HiRISE, and CTX images. The interpreted part of the CaSSIS images shown on (2B) and (2C) are indicated with white stripes. The background is the THEMIS Day image mosaic draped over the HRSC-MOLA blended DEM. (5B) Interpretation of part of CaSSIS image MY37_023188_115_0 (filters NIR-PAN-BLU as RGB). The contours are from the MOLA MEGDR map 128ppd (88). Fissures and a graben are aligned along topographic highs underlined by dark halos and culminated at bright patches. (5C) Interpretation of part of CaSSIS image MY36_022031_116_0 (filters NIR-PAN-BLU as RGB). The contours are from the MOLA MEGDR map 128ppd (94). Contours are poorly constrained due to the paucity of MOLA tracks. Nevertheless, the contours emphasize a North-trending control of topography.

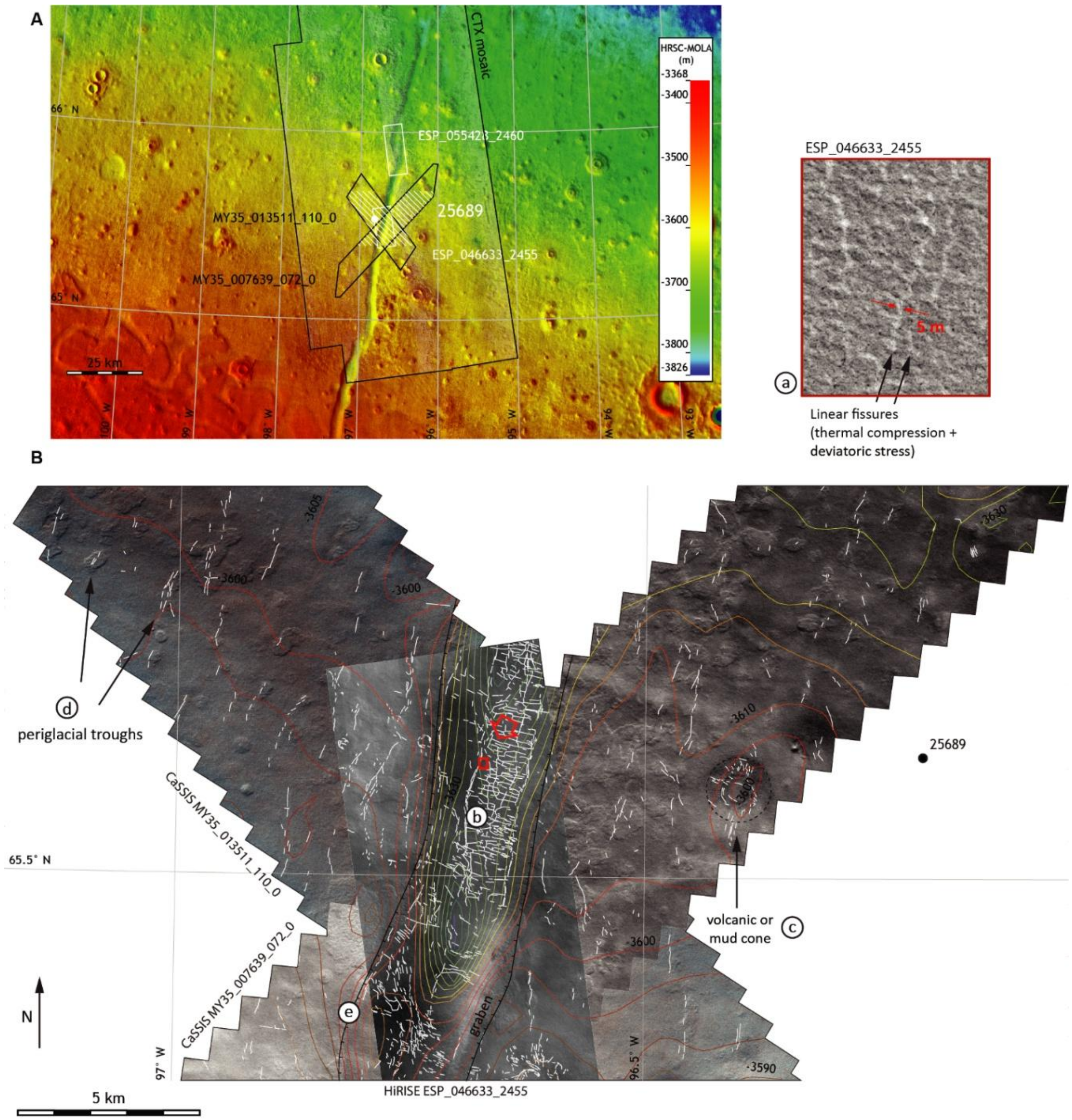

Fig. S6.

Interpretation of geology at Orbit 25689 ACS occultation. (6A) Location of the lowermost HCl detection by ACS (black dot); the analyzed CaSSIS, HiRISE, and CTX images, and (6B) (dashed area). The background is the THEMIS Day image mosaic draped over the HRSC MOLA blended DEM. (6B) Fissure map in the Orbit 25689 area. The analyzed dataset includes one HiRISE and two CaSSIS images. Only the fissures that denote influence of deviatoric stress (i.e., not related to permafrost activity forcing only) were mapped. The red arrow points toward a HiRISE zoom area (red box) area enlarged in the upper left. HiRISE image resolution, 0.5 m/pixel, is usually not enough to characterize fissure morphology as for Orbit 8886; however, this image shows that in some places, like for Orbit 8886, the dark-toned depression can be inferred between bright stripes. Circled letters are commented in the Supplementary Text.

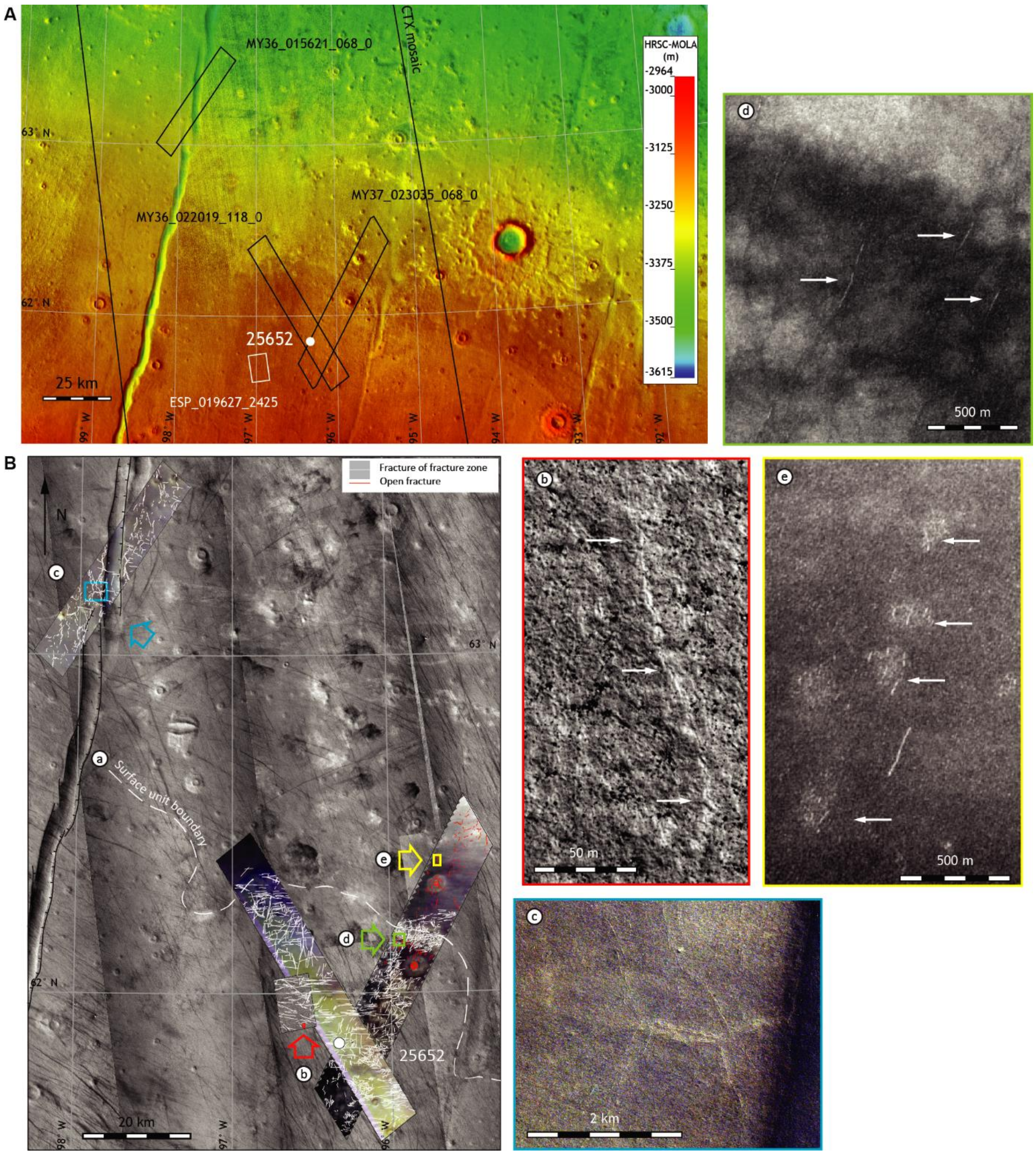

Fig. S7.

Interpretation of geology at Orbit 25652 ACS occultation. (7A) Location of the lowermost HCl detection by ACS (white dot); the analyzed CaSSIS, HiRISE, and CTX images, and fig. 7B (dashed area). The background is the THEMIS Day image mosaic draped over the HRSC MOLA blended DEM. (7B) Fissure map in the Orbit 25652 area. The analyzed dataset includes one HiRISE and three CaSSIS images. The background shows CTX images. Two distinct geologic units were found which were not identified in earlier works (a). Two fissured areas are enlarged in (b) and (c). They are interpreted as equivalent to the fissures seen on fig. S1. Fracture zones in (d) and (e) (white arrows) are perhaps similar to (b) and (c). Fractures are dominantly dark;

however, in (d), they are locally highlighted by bright patterns, which could be sites of ongoing active processes. In (e), the eroded dark LDM reveals an alignment of light patches (arrows) which is parallel to underlying bright fractures. This suggests that the fractures are interacted with the LDM, where ice is thought to be largely dominant (*60*), and contributed to their local erosion.

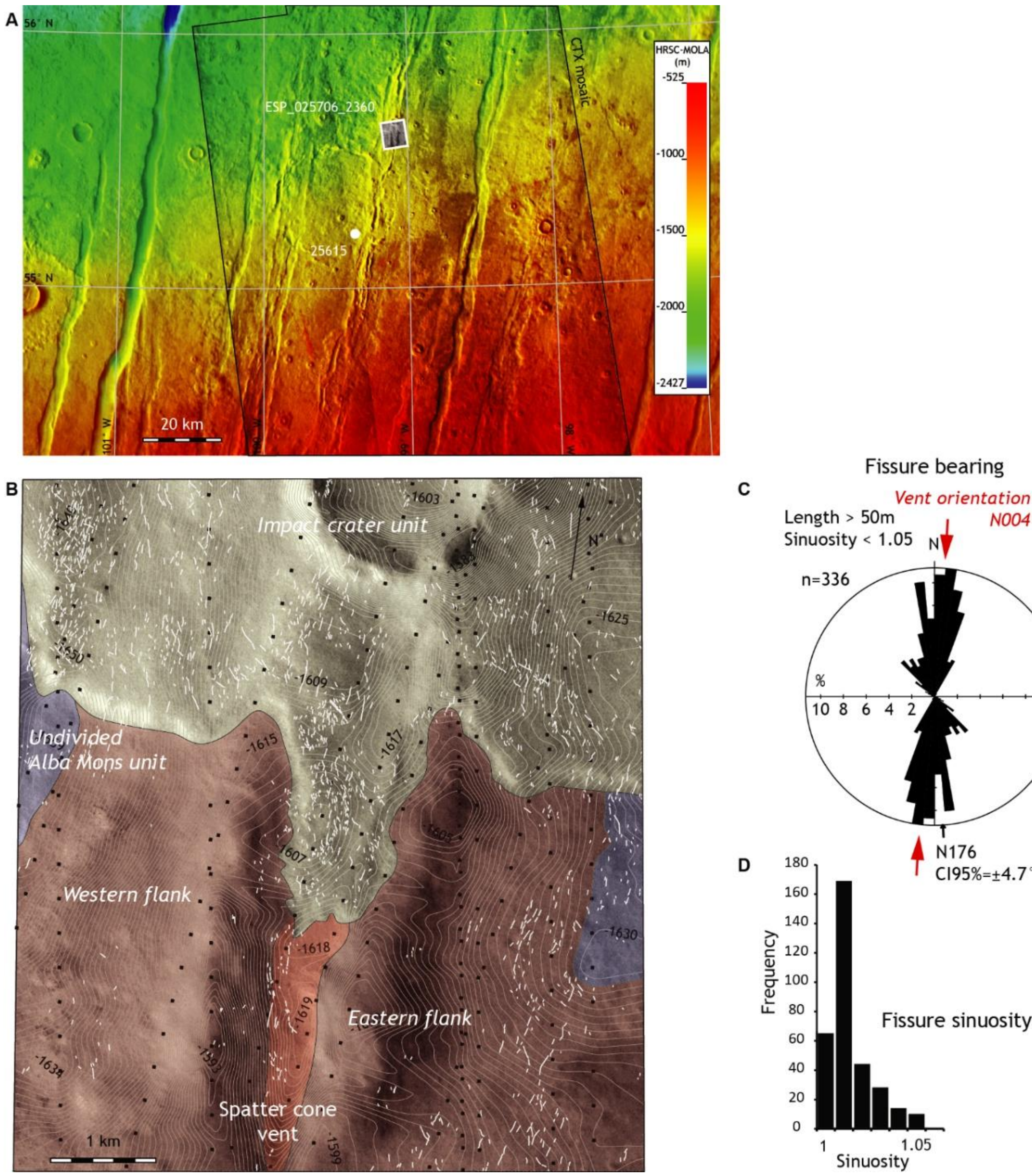

Fig. S8.

Interpretation of geology at Orbit 25615 ACS occultation. (8A) Location of the lowermost HCl detection by ACS (white dot) and the analyzed HiRISE and CTX images. The background is the THEMIS Day image mosaic draped over the HRSC MOLA blended DEM. (8B) Interpretation of the HiRISE image located on (8A). The white lines are fissures displaying one dark line framed by two bright stripes. Contours are interpolated from the MOLA shots (black dots). The rose diagram (8C) shows the orientation of the 336 fissures (n) which are longer than 50 m and have a sinuosity lesser than 1.05 (8D), which allows to filter those potentially influenced by processes involved in periglacial polygon evolution. The mean orientation of the fissures, N176 (CI = Confidence interval), is close to the N004 orientation of the spatter rampart, suggesting reactivation of rampart structures after impact crater formation and LDM deposition.

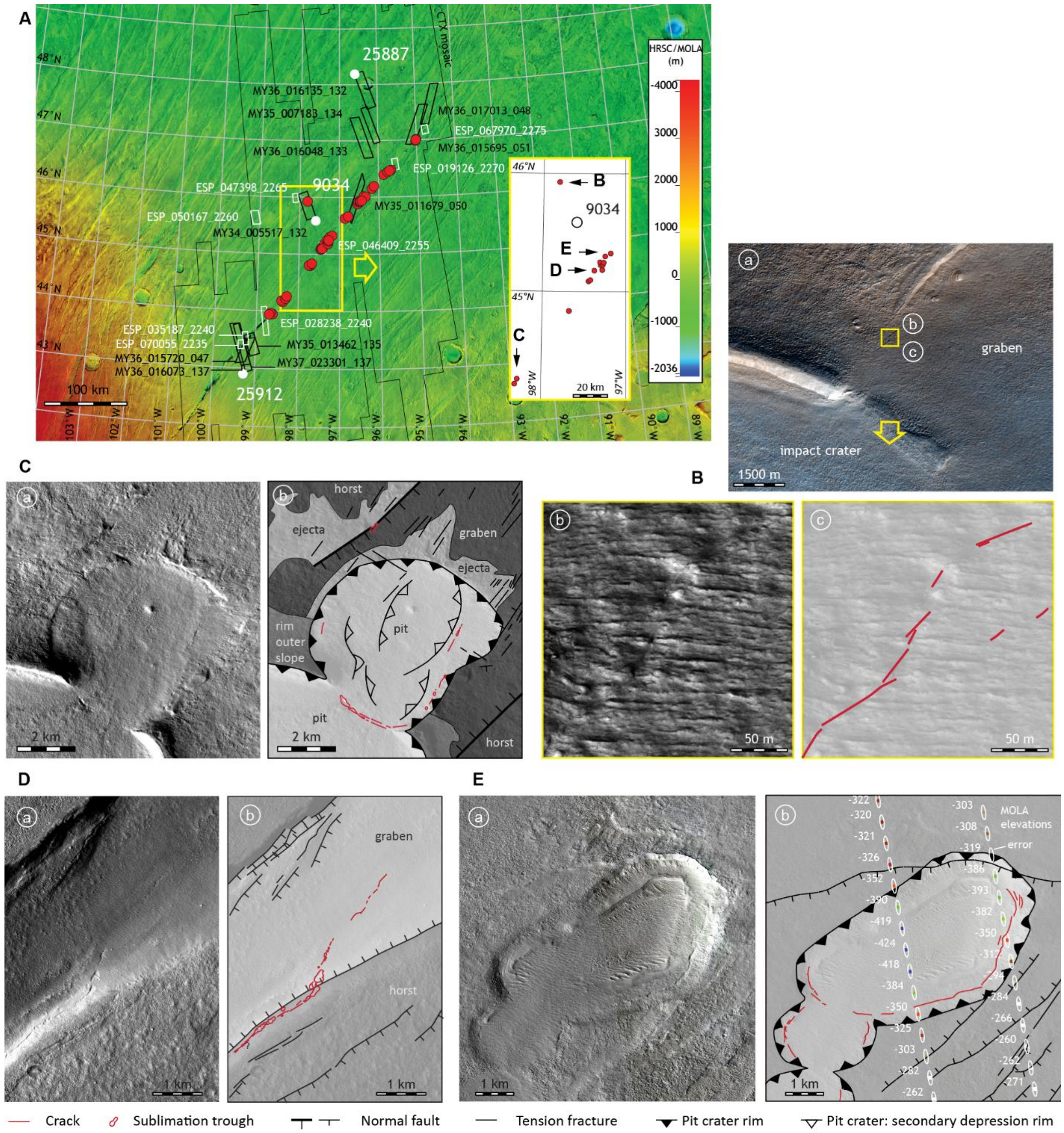

Fig. S9.

Interpretation of geology at ACS occultation orbits 25887, 9034, and 25912. (9A). Orbits 25887, 9034 and 25912: Location of the lowermost HCl detection by ACS (white dot) and the analyzed HiRISE and CTX images. The background is the THEMIS Day image mosaic draped over the HRSC MOLA blended DEM. The red dots locate the cracks which affect the LDM in the Phlegethon Catena pits and grabens. (9B) Post latitude-dependent mantle *en échelon* fissures affecting LDM in Phlegeton Catena, in the vicinity of ACS detection made at orbit 9034. (a) CaSSIS image MY34_005517_132_0; RGB product. (b) High-resolution of part of the *en échelon* fissure; HiRISE image ESP_082648_2260. (c) Fissure segment mapping. (9C) Post-latitude-dependent mantle deformation and sublimation patterns in Phlegeton Catena, at the

vicinity of ACS detection made at orbit 9034. (a) CTX image D21_035332_2260_XN_46N098W. (b) Interpretation. The fracture of the southern rim of the pit was reactivated and displays sublimation troughs. (9D) Post-latitude-dependent mantle deformation and sublimation patterns in Phlegeton Catena, in the vicinity of ACS detection made at orbit 9034. (a) CTX image D21_035332_2260_XN_46N098W. (b) Interpretation. (9E) Post-latitude-dependent mantle deformation and sublimation patterns in Phlegeton Catena, at the vicinity of ACS detection made at orbit 9034. (a) HiRISE image ESP_046409_2255. (b) Interpretation. The points give MOLA shot elevations; color is proportional to elevation and the ellipse provides uncertainty on geographic location. The cracks and sublimation troughs are located at mid-slope. Crack and trough overall geometry suggests reactivation pit-forming faults. In (9B-E), cracks and sublimation troughs are interpreted as possible seepage structures.

REFERENCES

1. T. E. Graedel, W. C. Keene, The budget and cycle of Earth's natural chlorine. *Pure Appl. Chem.* **68**, 1689–1697 (1996).

2. J. Filiberto, D. Baratoux, D. Beaty, D. Breuer, B. Farcy, M. Grott, J. Jones, W. Kiefer, P. Mane, F. McCubbin, S. Schwenzer, A review of volatiles in the Martian interior. *Meteorit. Planet. Sci.* **51**, 1935–1958 (2016).

3. A. Jackson, A. F. Davila, J. K. Böhlke, N. C. Sturchio, R. Sevanthi, N. Estrada, M. Brundrett, D. Lacelle, C. P. McKay, A. Poghosyan, W. Pollard, K. Zacny, Deposition, accumulation, and alteration of Cl^-, NO_3^-, ClO_4^- and ClO_3^- salts in a hyper-arid polar environment: Mass balance and isotopic constraints. *Geochim. Cosmochim. Acta* **182**, 197–215 (2016).

4. M. Luginin, A. Trokhimovskiy, B. Taysum, A. A. Fedorova, O. Korablev, K. S. Olsen, F. Montmessin, F. Lefèvre, Evidence of rapid hydrogen chloride uptake on water ice in the atmosphere of Mars. *Icarus* **411**, 115866 (2024).

5. G. Neukum, R. Jaumann, H. Hoffmann, E. Hauber, J. W. Head, A. T. Basilevsky, B. A. Ivanov, S. C. Werner, S. van Gasselt, J. B. Murray, T. McCord, Recent and episodic volcanic and glacial activity on Mars revealed by the high resolution stereo camera. *Nature* **432**, 971–979 (2004).

6. J. A. Richardson, J. A. Wilson, C. B. Connor, J. E. Bleacher, K. Kiyosugi, Recurrence rate and magma effusion rate for the latest volcanism on Arsia Mons, Mars. *Earth Planet. Sci. Lett.* **458**, 170–178 (2017).

7. E. Hauber, P. Brož, F. Jagert, P. Jodłowski, T. Platz, Very recent and wide-spread basaltic volcanism on Mars. *Geophys. Res. Lett.* **38**, L10201 (2011).

8. B. Pieterek, T. J. Jones, Mildly explosive eruptions at Martian low-shield volcanoes. *Commun. Earth Environ.* **5**, 542 (2024).

9. B. Pieterek, P. Brož, E. Hauber, Recent faulting at the Claritas Rupes scarp on Mars. *Icarus* **420**, 116198 (2024).

10. B. Pieterek, T. J. Jones, C. C. Ogbuagu, Late Amazonian-aged volcanic cones of explosive origin in Ceraunius Fossae, Tharsis, Mars. *Icarus* **445**, 116870 (2026).

11. G. P. Roberts, I. A. Crawford, D. Peacock, J. Vetterlein, E. Parfitt, L. Bishop, Possible evidence for on-going volcanism on Mars as suggested by thin, elliptical sheets of low-albedo particulate material around pits and fissures close to Cerberus Fossae. *Earth Moon Planets* **101**, 1–16 (2007).

12. M. Drilleau, H. Samuel, R. F. Garcia, A. Rivoldini, C. Perrin, M. Wieczorek, P. Lognonné, W. B. Banerdt, Constraints on lateral variations of Martian crustal thickness from seismological and gravity field measurements. *Geophys. Res. Lett.* **51**, e2023GL105701 (2024).

13. A.-C. Plesa, S. Padovan, N. Tosi, D. Breuer, M. Grott, M. A. Wieczorek, T. Spohn, S. E. Smrekar, W. B. Banerdt, The thermal state and interior structure of Mars. *Geophys. Res. Lett.* **45**, 12198–12209 (2018).

14. E. Hauber, D. Reiss, M. Ulrich, F. Preusker, F. Trauthan, M. Zanetti, H. Hiesinger, R. Jaumann, L. Johansson, A. Johnsson, S. Van Gasselt, M. Olvmo, Landscape evolution in Martian mid-latitude regions: Insights from analogous periglacial landforms in Svalbard. *Geol. Soc. Lond. Spec. Publ.* **356**, 111–131 (2011).

15. P. R. Christensen, J. L. Bandfield, J. F. Bell III, N. Gorelick, V. E. Hamilton, A. Ivanov, B. M. Jakosky, H. H. Kieffer, M. D. Lane, M. C. Malin, T. McConnochie, A. S. McEwen, H. Y. McSween Jr., G. L. Mehall, J. E. Moersch, K. H. Nealson, J. W. Rice Jr., M. I. Richardson, S. W. Ruff, M. D. Smith, T. N. Titus, M. B. Wyatt, Morphology and composition of the surface of Mars: Mars Odyssey THEMIS results. *Science* **300**, 2056–2061 (2003).

16. M. P. Milazzo, L. P. Keszthelyi, J. Radebaugh, A. G. Davies, E. P. Turtle, P. Geissler, K. P. Klaasen, J. A. Rathbun, A. S. McEwen, Volcanic activity at Tvashtar Catena, Io. *Icarus* **179**, 235–251 (2005).

17. T. Encrenaz, T. K. Greathouse, M. J. Richter, J. H. Lacy, T. Fouchet, B. Bézard, F. Lefèvre, F. Forget, S. K. Atreya, A stringent upper limit to SO2in the Martian atmosphere. *Astron. Astrophys.* **530**, A37 (2011).

18. V. A. Krasnopolsky, Search for methane and upper limits to ethane and SO_2 on Mars. *Icarus* **217**, 144–152 (2012).

19. A. S. J. Khayat, G. L. Villanueva, M. J. Mumma, A. T. Tokunaga, A deep search for the release of volcanic gases on Mars using ground-based high-resolution infrared and submillimeter spectroscopy: Sensitive upper limits for OCS and SO_2. *Icarus* **296**, 1–14 (2017).

20. I. A. Menyailov, Prediction of eruptions using changes in composition of volcanic gases. *Bull. Volcanol.* **39**, 112–125 (1975).

21. M. E. Zelenski, Y. A. Taran, Volcanic emissions of molecular chlorine. *Geochim. Cosmochim. Acta* **87**, 210–226 (2012).

22. R. B. Symonds, W. I. Rose, G. J. S. Bluth, T. M. Gerlach, Volcanic gas studies: Methods, results and applications. *Rev. Mineral. Geochem.* **30**, 1–66 (1994).

23. T. P. Fischer, Fluxes of volatiles (H_2O, CO_2, N_2, Cl, F) from arc volcanoes. *Geochem. J.* **42**, 21–38 (2008).

24. J. Filiberto, A. H. Treiman, Martian magmas contained abundant chlorine, but little water. *Geology* **37**, 1087–1090 (2009).

25. J. Filiberto, J. Gross, F. M. McCubbin, Constraints on the water, chlorine, and fluorine content of the Martian mantle. *Meteorit. Planet. Sci.* **51**, 2023–2035 (2016).

26. F. M. McCubbin, J. W. Boyce, P. Srinivasan, A. R. Santos, S. M. Elardo, J. Filiberto, A. Steele, C. K. Shearer, Heterogeneous distribution of H_2O in the Martian interior: Implications for the abundance of H_2O in depleted and enriched mantle sources. *Meteorit. Planet. Sci.* **51**, 2036–2060 (2016).

27. H. Wänke, G. Dreibus, Chemistry and accretion history of Mars. *Philos. Trans. R. Soc. Lond. Ser. A* **349**, 285–293 (1994).

28. O. Korablev, K. S. Olsen, A. Trokhimovskiy, F. Lefèvre, F. Montmessin, A. A. Fedorova, M. J. Toplis, J. Alday, D. A. Belyaev, A. Patrakeev, N. I. Ignatiev, A. V. Shakun, A. V. Grigoriev,

L. Baggio, I. Abdenour, G. Lacombe, Y. S. Ivanov, S. Aoki, I. R. Thomas, F. Daerden, B. Ristic, J. T. Erwin, M. Patel, G. Bellucci, J.-J. Lopez-Moreno, A. C. Vandaele, Transient HCl in the atmosphere of Mars. *Sci. Adv.* **7**, eabe4386 (2021).

29. O. Korablev, F. Montmessin, A. Trokhimovskiy, A. A. Fedorova, A. V. Shakun, A. V. Grigoriev, B. E. Moshkin, N. I. Ignatiev, F. Forget, F. Lefèvre, K. Anufreychik, I. Dzuban, Y. S. Ivanov, Y. K. Kalinnikov, T. O. Kozlova, A. Kungurov, V. Makarov, F. Martynovich, I. Maslov, D. Merzlyakov, P. P. Moiseev, Y. Nikolskiy, A. Patrakeev, D. Patsaev, A. Santos-Skripko, O. Sazonov, N. Semena, A. Semenov, V. Shashkin, A. Sidorov, A. V. Stepanov, I. Stupin, D. Timonin, A. Y. Titov, A. Viktorov, A. Zharkov, F. Altieri, G. Arnold, D. A. Belyaev, J. L. Bertaux, D. S. Betsis, N. Duxbury, T. Encrenaz, T. Fouchet, J.-C. Gérard, D. Grassi, S. Guerlet, P. Hartogh, Y. Kasaba, I. Khatuntsev, V. A. Krasnopolsky, R. O. Kuzmin, E. Lellouch, M. A. Lopez-Valverde, M. Luginin, A. Määttänen, E. Marcq, J. Martin Torres, A. S. Medvedev, E. Millour, K. S. Olsen, M. R. Patel, C. Quantin-Nataf, A. V. Rodin, V. I. Shematovich, I. Thomas, N. Thomas, L. Vazquez, M. Vincendon, V. Wilquet, C. F. Wilson, L. V. Zasova, L. M. Zelenyi, M. P. Zorzano, The Atmospheric Chemistry Suite (ACS) of three spectrometers for the ExoMars 2016 Trace Gas Orbiter. *Space Sci. Rev.* **214**, 7 (2018).

30. V. A. Krasnopolsky, G. L. Bjoraker, M. J. Mumma, D. E. Jennings, Jennings High-resolution spectroscopy of Mars at 3.7 and 8 µm: A sensitive search for H_2O_2, H_2CO, HCl, and CH_4, and detection of HDO. *J. Geophys. Res. Planets* **102**, E3 (1997).

31. P. Hartogh, C. Jarchow, E. Lellouch, M. de Val-Borro, M. Rengel, R. Moreno, A. S. Medvedev, H. Sagawa, B. M. Swinyard, T. Cavalié, D. C. Lis, M. I. Błęcka, M. Banaszkiewicz, D. Bockelée-Morvan, J. Crovisier, T. Encrenaz, M. Küppers, L.-M. Lara, S. Szutowicz, B. Vandenbussche, F. Bensch, E. A. Bergin, F. Billebaud, N. Biver, G. A. Blake, J. A. D. L. Blommaert, J. Cernicharo, L. Decin, P. Encrenaz, H. Feuchtgruber, T. Fulton, T. de Graauw, E. Jehin, M. Kidger, R. Lorente, D. A. Naylor, G. Portyankina, M. Sánchez-Portal, R. Schieder, S. Sidher, N. Thomas, E. Verdugo, C. Waelkens, N. Whyborn, D. Teyssier, F. Helmich, P. Roelfsema, J. Stutzki, H. G. LeDuc, J. A. Stern, Herschel/HIFI observations of Mars: First detection of O_2 at submillimetre wavelengths and upper limits on HCl and H_2O_2. *Astron. Astrophys.* **521**, L49 (2010).

32. G. L. Villanueva, M. J. Mumma, R. E. Novak, Y. L. Radeva, H. U. Käufl, A. Smette, A. Tokunaga, A. Khayat, T. Encrenaz, P. Hartogh, A sensitive search for organics (CH_4, CH_3OH, H_2CO, C_2H_6, C_2H_2, C_2H_4), hydroperoxyl (HO_2), nitrogen compounds (N_2O, NH_3, HCN) and chlorine species (HCl, CH_3Cl) on Mars using ground-based high-resolution infrared spectroscopy. *Icarus* **223**, 11–27 (2013).

33. S. Aoki, F. Daerden, S. Viscardy, I. R. Thomas, J. T. Erwin, S. Robert, L. Trompet, L. Neary, G. L. Villanueva, G. Liuzzi, M. M. J. Crismani, R. T. Clancy, J. Whiteway, F. Schmidt, M. A. Lopez-Valverde, B. Ristic, M. R. Patel, G. Bellucci, J.-J. Lopez-Moreno, K. S. Olsen, F. Lefèvre, F. Montmessin, A. Trokhimovskiy, A. A. Fedorova, O. Korablev, A. C. Vandaele, Annual appearance of hydrogen chloride on Mars and a striking similarity with the water vapor vertical distribution observed by TGO/NOMAD. *Geophys. Res. Lett.* **48**, e2021GL092506 (2021).

34. K. S. Olsen, A. Trokhimovskiy, L. Montabone, A. A. Fedorova, M. Luginin, F. Lefèvre, O. I. Korablev, F. Montmessin, F. Forget, E. Millour, A. Bierjon, L. Baggio, J. Alday, C. F. Wilson, P. G. J. Irwin, D. A. Belyaev, A. Patrakeev, A. Shakun, Seasonal reappearance of HCl in the atmosphere of Mars during the Mars year 35 dusty season. *Astron. Astrophys.* **647**, A161 (2021).

35. K. S. Olsen, A. A. Fedorova, D. M. Kass, A. Kleinböhl, A. Trokhimovskiy, O. I. Korablev, F. Montmessin, F. Lefèvre, L. Baggio, J. Alday, D. A. Belyaev, J. A. Holmes, J. P. Mason, P. M. Streeter, K. Rajendran, M. R. Patel, A. Patrakeev, A. Shakun, Relationships between HCl, H_2O, aerosols, and temperature in the Martian atmosphere: 1. Climatological Outlook. *J. Geophys. Res. Planets.* **129**, e2024JE008350 (2024).

36. D. M. Kass, A. Kleinböhl, D. J. McCleese, J. T. Schofield, M. D. Smith, Interannual similarity in the Martian atmosphere during the dust storm season. *Geophys. Res. Lett.* **43**, 6111–6118 (2016).

37. A. A. Fedorova, F. Montmessin, O. Korablev, M. Luginin, A. Trokhimovskiy, D. A. Belyaev, N. I. Ignatiev, F. Lefèvre, J. Alday, P. G. J. Irwin, K. S. Olsen, J.-L. Bertaux, E. Millour, A. Määttänen, A. Shakun, A. V. Grigoriev, A. Patrakeev, S. Korsa, N. Kokonkov, L. Baggio, F.

Forget, C. F. Wilson, Stormy water on Mars: The distribution and saturation of atmospheric water during the dusty season. *Science* **367**, 297–300 (2020).

38. A. Trokhimovskiy, A. A. Fedorova, K. S. Olsen, J. Alday, O. Korablev, F. Montmessin, F. Lefèvre, A. Patrakeev, D. Belyaev, A. V. Shakun, Isotopes of chlorine from HCl in the Martian atmosphere. *Astron. Astrophys.* **651**, A32 (2021).

39. K. A. Farley, P. Martin, P. D. Archer, S. K. Atreya, P. G. Conrad, J. L. Eigenbrode, A. G. Fairén, H. B. Franz, C. Freissinet, D. P. Glavin, P. R. Mahaffy, C. Malespin, D. W. Ming, R. Navarro-Gonzalez, B. Sutter, Light and variable 37 Cl/ 35 Cl ratios in rocks from Gale Crater, Mars: Possible signature of perchlorate. *Earth Planet. Sci. Lett.* **438**, 14–24 (2016).

40. V. A. Krasnopolsky, Photochemistry of HCl in the Martian atmosphere. *Icarus* **374**, 114807 (2022).

41. P. M. Streeter, K. Rajendran, S. Lewis, K. S. Olsen, A. Trokhimovskiy, O. I. Korablev, M. R. Patel, Global distribution and seasonality of Martian atmospheric HCl explained through heterogeneous chemistry. *Geophys. Res. Lett.* **52**, e2024GL111059 (2025).

42. B. M. Taysum, P. I. Palmer, K. Olsen, M. Luginin, N. Ignatiev, A. Trokhimovskiy, A. Shakun, A. V. Grigoriev, F. Montmessin, O. Korablev, Observed seasonal changes in Martian hydrogen chloride explained by heterogeneous chemistry. *Astron. Astrophys.* **687**, A191 (2024).

43. K. S. Olsen, A. A. Fedorova, D. M. Kass, A. Kleinböhl, A. Trokhimovskiy, O. I. Korablev, F. Montmessin, F. Lefèvre, L. Baggio, J. Alday, D. A. Belyaev, J. A. Holmes, J. P. Mason, P. M. Streeter, K. Rajendran, M. R. Patel, A. Patrakeev, A. Shakun, Relationships between HCl, H_2O, aerosols, and temperature in the Martian atmosphere: 2. Quantitative Correlations. *J. Geophys. Res. Planets* **129**, e2024JE008351 (2024).

44. R. T. Clancy, F. Montmessin, J. Benson, F. Daerden, A. Colaprete, M. J. Wolff, "Mars clouds," in *The Atmosphere and Climate of Mars*, Cambridge Planetary Science , R. M. Haberle, R. T. Clancy, F. Forget, M. D. Smith, R. W. Zurek, Eds. (Cambridge Univ. Press, 2017), pp. 76–105.

45. Z. Wu, T. Li, J. Li, C. Yang, J. Cui, Diurnal variations of water ice in the Martian atmosphere observed by Mars Climate Sounder. *Remote Sens.* **14**, 2235 (2022).

46. F. Montmessin, M. D. Smith, Y. Langevin, M. T. Mellon, A. A. Fedorova, “The water cycle,” in *The Atmosphere and Climate of Mars*, Cambridge Planetary Science, R. M. Haberle, R. T. Clancy, F. Forget, M. D. Smith, R. W. Zurek, Eds. (Cambridge Univ. Press, 2017), pp. 338–373.

47. P. Brož, D. Oehler, A. Mazzini, E. Hauber, G. Komatsu, G. Etiope, V. Cuřín, An overview of sedimentary volcanism on Mars. *Earth Surf. Dyn.* **11**, 633–661 (2023).

48. J. Clark, B. Sutter, P. D. Archer Jr., D. Ming, E. Rampe, A. McAdam, R. Navarro-González, J. Eigenbrode, D. Glavin, M.-P. Zorzano, J. Martin-Torres, R. Morris, V. Tu, S. J. Ralston, P. Mahaffy, A review of sample analysis at Mars-evolved gas analysis laboratory analog work supporting the presence of perchlorates and chlorates in Gale crater, Mars. *Minerals* **11**, 475 (2021).

49. K. L. Tanaka, C. M. Fortezzo, R. K. Hayward, J. A. P. Rodriguez, J. A. Skinner Jr., History of plains resurfacing in the Scandia region of Mars. *Planet. Space Sci.* **59**, 1128–1142 (2011).

50. H.-C. Chao, C.-F. You, I.-T. Lin, H.-C. Liu, L.-H. Chung, C.-C. Huang, C.-H. Chung, Two-end-member mixing in the fluids emitted from mud volcano Lei-Gong-Huo, Eastern Taiwan: Evidence from Sr isotopes. *Front. Earth Sci.* **9**, 750436 (2022).

51. J. Binti Yussibnosh, M. V. Prasanna, N. K. Modon Valappil, Geochemical evaluation of mud volcanic sediment and water in Northern Borneo: A baseline study. *Total Environ. Res. Themes* **6**, 100033 (2023).

52. M. H. Hecht, S. P. Kounaves, R. C. Quinn, S. J. West, S. M. M. Young, D. W. Ming, D. C. Catling, B. C. Clark, W. V. Boynton, J. Hoffman, L. P. DeFlores, K. Gospodinova, J. Kapit, P. H. Smith, Detection of perchlorate and the soluble chemistry of Martian soil at the Phoenix lander site. *Science* **325**, 64–67 (2009).

53. B. C. Clark, S. P. Kounaves, Evidence for the distribution of perchlorates on Mars. *Int. J. Astrobiol.* **15**, 311–318 (2016).

54. M. R. Salvatore, P. R. Christensen, On the origin of the Vastitas Borealis Formation in Chryse and Acidalia Planitiae, Mars. *J. Geophys. Res. Planets* **119**, 2437–2456 (2014).

55. S. C. Werner, K. L. Tanaka, J. A. Skinner Jr., Mars: The evolutionary history of the northern lowlands based on crater counting and geologic mapping. *Planet. Space Sci.* **59**, 1143–1165 (2011).

56. S. Wiedeking, A. Lentz, J. H. Pasckert, J. Raack, N. Schmedemann, H. Hiesinger, Rheological properties and ages of lava flows on Alba Mons, Mars. *Icarus* **389**, 115267 (2023).

57. D. Mège, P. Masson, A plume tectonics model for the Tharsis province, Mars. *Planet. Space Sci.* **44**, 1499–1546 (1996).

58. S. Zhong, Migration of Tharsis volcanism on Mars caused by differential rotation of the lithosphere. *Nat. Geosci.* **2**, 19–23 (2009).

59. M. T. Mellon, R. E. Arvidson, H. G. Sizemore, M. L. Searls, D. L. Blaney, S. Cull, M. H. Hecht, T. L. Heet, H. U. Keller, M. T. Lemmon, W. J. Markiewicz, D. W. Ming, R. V. Morris, W. T. Pike, A. P. Zent, Ground ice at the Phoenix Landing Site: Stability state and origin. *J. Geophys. Res. Planets* **114**, (2009).

60. J. S. Levy, J. W. Head, D. R. Marchant, Gullies, polygons and mantles in Martian permafrost environments: Cold desert landforms and sedimentary processes during recent Martian geological history. *Geol. Soc. Lond. Spec. Publ.* **354**, 167–182 (2011).

61. E. Vos, F. Forget, L. Lange, J. Naar, J. B. Clement, E. Millour, The Martian mid-latitude subsurface ice is the remnant of a past ice sheet. *Commun. Earth Environ.* **7**, 412 (2026).

62. J. D. Ramsdale, M. R. Balme, S. J. Conway, C. Gallagher, S. A. van Gasselt, E. Hauber, C. Orgel, A. Séjourné, J. A. Skinner, F. Costard, A. Johnsson, A. Losiak, D. Reiss, Z. M. Swirad, A. Kereszturi, I. B. Smith, T. Platz, Grid-based mapping: A method for rapidly determining

the spatial distributions of small features over very large areas. *Planet. Space Sci.* **140**, 49–61 (2017).

63. J. D. Ramsdale, M. R. Balme, C. Gallagher, S. J. Conway, I. B. Smith, E. Hauber, C. Orgel, A. Séjourné, F. Costard, V. R. Eke, S. A. van Gasselt, A. Johnsson, Á. Kereszturi, A. Łosiak, R. J. Massey, T. Platz, D. Reiss, Z. M. Swirad, L. F. A. Teodoro, J. T. Wilson, Grid mapping the northern plains of Mars: Geomorphological, radar, and water-equivalent hydrogen results from Arcadia Plantia. *J. Geophys. Res. Planets* **124**, 504–527 (2019).

64. C. Orgel, E. Hauber, S. van Gasselt, D. Reiss, A. Johnsson, J. D. Ramsdale, I. Smith, Z. M. Swirad, A. Séjourné, J. T. Wilson, M. R. Balme, S. J. Conway, F. Costard, V. R. Eke, C. Gallagher, Á. Kereszturi, A. Łosiak, R. J. Massey, T. Platz, J. A. Skinner, L. F. A. Teodoro, Grid mapping the northern plains of Mars: A new overview of recent water- and ice-related landforms in Acidalia Planitia. *J. Geophys. Res. Planets* **124**, 454–482 (2019).

65. F. Séjourné, Z. M. Costard, A. Swirad, S. Łosiak, I. Bouley, M. R. Smith, C. Balme, J. D. Orgel, E. Ramsdale, S. J. Hauber, S. Conway, D. van Gasselt, A. Reiss, C. Johnsson, J. A. Gallagher, Á. Skinner, T. Kereszturi, T. Platz, Grid mapping the northern plains of Mars: Using morphotype and distribution of ice-related landforms to understand multiple ice-rich deposits in Utopia Planitia. *J. Geophys. Res. Planets* **124**, 483–503 (2019).

66. H. S. Bertelsen, F. Guldstrand, F. Sigmundsson, R. Pedersen, K. Mair, O. Galland, Beyond elasticity: Are Coulomb properties of the Earth's crust important for volcano geodesy? *J. Volcanol. Geotherm. Res.* **410**, 107153 (2021).

67. D. C. Catling, M. W. Claire, K. J. Zahnle, R. C. Quinn, B. C. Clark, M. H. Hecht, S. Kounaves, Atmospheric origins of perchlorate on Mars and in the Atacama. *J. Geophys. Res. Planets* **115**, (2010).

68. M. Edmonds, D. Pyle, C. Oppenheimer, HCl emissions at Soufrière Hills volcano, Montserrat, West Indies, during a second phase of dome building: November 1999 to October 2000. *Bull. Volcanol.* **64**, 21–30 (2002).

69. S. A. Carn, L. Clarisse, A. J. Prata, Multi-decadal satellite measurements of global volcanic degassing. *J. Volcanol. Geotherm. Res.* **311**, 99–134 (2016).

70. A. Riddell, M. Burton, B. Esse, B. McCormick Kilbride, A. Chiarugi, T. Christopher, F. D'Amato, G. A. Ryan, A. Stinton, S. Viciani, Quantification of low-temperature gas emissions reveals CO_2 flux underestimates at Soufrière Hills volcano, Montserrat. *Sci. Adv.* **11**, eads8864 (2025).

71. S. S. Hamid, L. Kerber, A. B. Clarke, F. Forget, The delivery of water ice to the Martian surface by passive degassing. *Icarus* **416**, 116084 (2024).

72. M. L. Smith, M. W. Claire, D. C. Catling, K. J. Zahnle, The formation of sulfate, nitrate and perchlorate salts in the Martian atmosphere. *Icarus* **231**, 51–64 (2014).

73. S. Aoki, S. Faggi, G. L. Villanueva, G. Liuzzi, H. Sagawa, F. Daerden, S. Viscardy, S. Koyama, A. C. Vandaele, Global mapping of HCl on Mars by IRTF/iSHELL. *Planet. Sci. J.* **5**, 158 (2024).

74. S. Faggi, S. Aoki, G. Liuzzi, G. Villanueva, H. Sagawa, M. J. Mumma, A. C. Vandaele, F. Daerden, S. Viscardy, Following the HCl cycle over three Martian seasons in Mars year 36. *J. Geophys. Res. Planets* **130**, e2025JE009105 (2025).

75. B. Benne, P. I. Palmer, B. M. Taysum, K. S. Olsen, F. Lefevre, A 3D model simulation of hydrogen chloride photochemistry on Mars: Comparison with satellite data. *Astron. Astrophys.* **699**, A362 (2025).

76. K. Rajendran, P. M. Streeter, S. R. Lewis, M. K. D. Duffy, J. A. Holmes, K. S. Olsen, O. Korablev, M. R. Patel, Global transport of chlorine species in the Martian atmosphere and the resulting surface distribution of perchlorates. *J. Geophys. Res. Planets* **130**, e2024JE008537 (2025).

77. G. Liuzzi, G. L. Villanueva, S. Viscardy, D. Mège, M. M. J. Crismani, S. Aoki, J. Gurgurewicz, P.-A. Tesson, M. J. Mumma, M. D. Smith, S. Faggi, V. Kofman, E. W. Knutsen, F. Daerden, L. Neary, F. Schmidt, L. Trompet, J. T. Erwin, S. Robert, I. R. Thomas,

B. Ristic, G. Bellucci, J. J. Lopez-Moreno, M. R. Patel, A. C. Vandaele, Probing the atmospheric Cl isotopic ratio on Mars: Implications for planetary evolution and atmospheric chemistry. *Geophys. Res. Lett.* **48**, e2021GL092650 (2021).

78. S. Guerlet, N. Ignatiev, F. Forget, T. Fouchet, P. Vlasov, G. Bergeron, R. M. B. Young, E. Millour, S. Fan, H. Tran, A. Shakun, A. Grigoriev, A. Trokhimovskiy, F. Montmessin, O. Korablev, Thermal structure and aerosols in Mars' atmosphere from TIRVIM/ACS onboard the ExoMars Trace Gas Orbiter: Validation of the retrieval algorithm. *J. Geophys. Res. Planets* **127**, e2021JE007062 (2022).

79. P. Vlasov, N. Ignatiev, S. Guerlet, D. Grassi, O. Korablev, A. Grigoriev, A. Shakun, D. Patsaev, I. Maslov, L. Zasova, M. Luginin, A. Trokhimovskiy, E. Millour, F. Forget, R. Haus, G. Arnold, F. Montmessin, Martian atmospheric thermal structure and dust distribution during the MY 34 global dust storm from ACS TIRVIM nadir observations. *J. Geophys. Res. Planets* **127**, e2022JE007272 (2022).

80. D. Mège, A. C. Cook, E. Garel, Y. Lagabrielle, M.-H. Cormier, Volcanic rifting at Martian grabens. *J. Geophys. Res. Planets* **108**, 5044 (2003).

81. R. Arvidson, D. Adams, G. Bonfiglio, P. Christensen, S. Cull, M. Golombek, J. Guinn, E. Guinness, T. Heet, R. Kirk, A. Knudson, M. Malin, M. Mellon, A. McEwen, A. Mushkin, T. Parker, F. Seelos IV, K. Seelos, P. Smith, D. Spencer, T. Stein, L. Tamppari, Mars Exploration Program 2007 Phoenix landing site selection and characteristics. *J. Geophys. Res. Planets* **113**, (2008).

82. M. T. Mellon, R. E. Arvidson, J. J. Marlow, R. J. Phillips, E. Asphaug, Periglacial landforms at the Phoenix landing site and the northern plains of Mars. *J. Geophys. Res. Planets* **113**, (2008).

83. A. S. McEwen, E. M. Eliason, J. W. Bergstrom, N. T. Bridges, C. J. Hansen, W. A. Delamere, J. A. Grant, V. C. Gulick, K. E. Herkenhoff, L. Keszthelyi, R. L. Kirk, M. T. Mellon, S. W. Squyres, N. Thomas, C. M. Weitz, Mars Reconnaissance Orbiter's High Resolution Imaging Science Experiment (HiRISE). *J. Geophys. Res. Planets* **112**, (2007).

84. N. Thomas, G. Cremonese, R. Ziethe, M. Gerber, M. Brändli, G. Bruno, M. Erismann, L. Gambicorti, T. Gerber, K. Ghose, M. Gruber, P. Gubler, H. Mischler, J. Jost, D. Piazza, A. Pommerol, M. Rieder, V. Roloff, A. Servonet, W. Trottmann, T. Uthaicharoenpong, C. Zimmermann, D. Vernani, M. Johnson, E. Pelò, T. Weigel, J. Viertl, N. De Roux, P. Lochmatter, G. Sutter, A. Casciello, T. Hausner, I. Ficai Veltroni, V. Da Deppo, P. Orleanski, W. Nowosielski, T. Zawistowski, S. Szalai, B. Sodor, S. Tulyakov, G. Troznai, M. Banaskiewicz, J. C. Bridges, S. Byrne, S. Debei, M. R. El-Maarry, E. Hauber, C. J. Hansen, A. Ivanov, L. Keszthelyi, R. Kirk, R. Kuzmin, N. Mangold, L. Marinangeli, W. J. Markiewicz, M. Massironi, A. S. McEwen, C. Okubo, L. L. Tornabene, P. Wajer, J. J. Wray, The Colour and Stereo Surface Imaging System (CaSSIS) for the ExoMars Trace Gas Orbiter. *Space Sci. Rev.* **212**, 1897–1944 (2017).

85. M. C. Malin, J. F. Bell III, B. A. Cantor, M. A. Caplinger, W. M. Calvin, R. T. Clancy, K. S. Edgett, L. Edwards, R. M. Haberle, P. B. James, S. W. Lee, M. A. Ravine, P. C. Thomas, M. J. Wolff, Context Camera Investigation on board the Mars Reconnaissance Orbiter. *J. Geophys. Res. Planets* **112**, (2007).

86. P. R. Christensen, E. Engle, S. Anwar, S. Dickenshied, D. Noss, N. Gorelick, M. Weiss-Malik, “JMARS—A planetary GIS,” Abstract in AGU Fall Meeting Abstracts (2009); http://adsabs.harvard.edu/abs/2009AGUFMIN22A..06C.

87. D. Smith, M. T. Zuber, G. A. Neumann, P. Jester, Mars global surveyor laser altimeter precision experiment data record, MGS-M-MOLA-3-PEDR-L1A-V1.0, NASA Planetary Data System (2003); https://doi.org/10.17189/1519520.

88. C. Re, A. Fennema, E. Simioni, S. Sutton, D. Mège, K. Gwinner, M. Józefowicz, G. Munaretto, M. Pajola, A. Petrella, A. Pommerol, G. Cremonese, N. Thomas, CaSSIS-based stereo products for Mars after three years in orbit. *Planet. Space Sci.* **219**, 105515 (2022).

89. K. L. Tanaka, J. A. Skinner Jr., J. M. Dohm, R. P. Irwin III, E. J. Kolb, C. M. Fortezzo, T. Platz, G. G. Michael, T. M. Hare, Geologic map of Mars: U.S. Geological Survey Scientific Investigations Map 3292, scale 1:20,000,000 (2014); https://doi.org/10.3133/sim3292.

90. R. L Fergason, T. M. Hare, J. Laura, HRSC and MOLA Blended Digital Elevation Model at 200m v2. Astrogeology PDS Annex, U.S. Geological Survey (2018); https://astrogeology.usgs.gov/search/map/mars_mgs_mola_mex_hrsc_blended_dem_global_200m.

91. M. M. Abdelmalak, R. Mourgues, O. Galland, D. Bureau, Fracture mode analysis and related surface deformation during dyke intrusion: Results from 2D experimental modelling. *Earth Planet. Sci. Lett.* **359-360**, 93–105 (2012).

92. J. S. Levy, D. R. Marchant, J. W. Head, Thermal contraction crack polygons on Mars: A synthesis from HiRISE, Phoenix, and terrestrial analog studies. *Icarus* **206**, 229–252 (2010).

93. D. E. Smith, M. T. Zuber, G. A. Neumann, E. A. Guinness, S. Slavney, Mars global surveyor laser altimeter mission experiment gridded data record, MGS-M-MOLA-5-MEGDR-L3-V1.0, NASA Planetary Data System (2003); https://doi.org/10.17189/1519460.

94. C. S. Edwards, K. J. Nowicki, P. R. Christensen, J. Hill, N. Gorelick, K. Murray, Mosaicking of global planetary image datasets: 1. Techniques and data processing for Thermal Emission Imaging System (THEMIS) multi-spectral data. *J. Geophys. Res. Planets* **116**, E10008 (2011).

95. G. J. Taylor, The bulk composition of Mars. *Geochemistry* **73**, 401–420 (2013).

96. J. M. Keller, W. V. Boynton, S. Karunatillake, V. R. Baker, J. M. Dohm, L. G. Evans, M. J. Finch, B. C. Hahn, D. K. Hamara, D. M. Janes, K. E. Kerry, H. E. Newsom, R. C. Reedy, A. L. Sprague, S. W. Squyres, R. D. Starr, G. J. Taylor, R. M. S. Williams, Equatorial and midlatitude distribution of chlorine measured by Mars Odyssey GRS. *J. Geophys. Res. Planets* **111**, (2006).

97. W. V. Boynton, G. J. Taylor, L. G. Evans, R. C. Reedy, R. Starr, D. M. Janes, K. E. Kerry, D. M. Drake, K. J. Kim, R. M. S. Williams, M. K. Crombie, J. M. Dohm, V. Baker, A. E. Metzger, S. Karunatillake, J. M. Keller, H. E. Newsom, J. R. Arnold, J. Brückner, P. A. J. Englert, O. Gasnault, A. L. Sprague, I. Mitrofanov, S. W. Squyres, J. I. Trombka, L. d'Uston, H. Wänke, D. K. Hamara, Concentration of H, Si, Cl, K, Fe, and Th in the low- and mid-latitude regions of Mars. *J. Geophys. Res. Planets* **112**, E12S99 (2007).

98. M. L. Litvak, I. G. Mitrofanov, R. Gellert, M. V. Djachkova, D. I. Lisov, A. R. Vasavada, S. Czarnecki, Depth distribution of chlorine at Gale crater, Mars, as derived from the DAN and APXS experiments onboard the curiosity rover. *J. Geophys. Res. Planets* **128**, e2022JE007694 (2023).

99. M. M. Osterloo, V. E. Hamilton, J. L. Bandfield, T. D. Glotch, A. M. Baldridge, P. R. Christensen, L. L. Tornabene, F. S. Anderson, Chloride-bearing materials in the southern highlands of Mars. *Science* **319**, 1651–1654 (2008).

100. J. A. Berger, M. E. Schmidt, R. Gellert, J. L. Campbell, P. L. King, R. L. Flemming, D. W. Ming, B. C. Clark, I. Pradler, S. J. V. VanBommel, M. E. Minitti, A. G. Fairén, N. I. Boyd, L. M. Thompson, G. M. Perrett, B. E. Elliott, E. Desouza, A global Mars dust composition refined by the alpha-particle x-ray spectrometer in Gale Crater. *Geophys. Res. Lett.* **43**, 67–75 (2016).

101. Z. Sharp, J. Williams, C. Shearer, C. Agee, K. McKeegan, The chlorine isotope composition of Martian meteorites 2. Implications for the early solar system and the formation of Mars. *Meteorit. Planet. Sci.* **51**, 2111–2126 (2016).

102. A. Wang, Y. Yan, B. L. Jolliff, S. M. McLennan, K. Wang, E. Shi, W. M. Farrell, Chlorine release from common chlorides by Martian dust activity. *J. Geophys. Res. Planets* **125**, e2019JE006283 (2020).

103. J. L. Grenfell, F. Wunderlich, M. Sinnhuber, K. Herbst, R. Lehmann, M. Scheucher, S. Gebauer, G. Arnold, H. Rauer, Atmospheric processes affecting methane on Mars. *Icarus* **382**, 114940 (2022).

104. F. Lefèvre, V. Krasnopolsky, in *The Atmosphere and Climate of Mars*, Cambridge Planetary Science, R. M. Haberle, R. T. Clancy, F. Forget, M. D, Smith, R. W. Zurek, Eds. (Cambridge Univ. Press, 2017), pp. 405–432.

105. M. Kippenberger, G. Schuster, J. Lelieveld, J. N. Crowley, Trapping of HCl and oxidised organic trace gases in growing ice at temperatures relevant to cirrus clouds. *Atmos. Chem. Phys.* **19**, 11939–11951 (2019).

106. S. Solomon, Stratospheric ozone depletion: A review of concepts and history. *Rev. Geophys.* **37**, 275–316 (1999).

107. B. S. Berland, M. A. Tolbert, S. M. George, Surface sensitive studies of the reactive uptake of chlorine nitrate on ice. *J. Phys. Chem. A* **101**, 9954–9963 (1997).

108. R. G. Hynes, J. C. Mössinger, R. A. Cox, The interaction of HCl with water-ice at tropospheric temperatures. *Geophys. Res. Lett.* **28**, 2827–2830 (2001).

109. J. Farquhar, J. Savarino, T. Jackson, M. H. Thiemens, Evidence of atmospheric sulphur in the Martian regolith from sulphur isotopes in meteorites. *Nature* **404**, 50–52 (2000).

110. P. L. King, S. M. McLennan, Sulfur on Mars. *Elements* **6**, 107–112 (2010).

111. F. Gaillard, B. Scaillet, The sulfur content of volcanic gases on Mars. *Earth Planet. Sci. Lett.* **279**, 34–43 (2009).

112. J.-P. Bibring, Y. Langevin, J. F. Mustard, F. Poulet, R. Arvidson, A. Gendrin, B. Gondet, N. Mangold, P. Pinet, F. Forget, M. Berthé, J.-P. Bibring, A. Gendrin, C. Gomez, B. Gondet, D. Jouglet, F. Poulet, A. Soufflot, M. Vincendon, M. Combes, P. Drossart, T. Encrenaz, T. Fouchet, R. Merchiorri, G. Belluci, F. Altieri, V. Formisano, F. Capaccioni, P. Cerroni, A. Coradini, S. Fonti, O. Korablev, V. Kottsov, N. Ignatiev, V. Moroz, D. Titov, L. Zasova, D. Loiseau, N. Mangold, P. Pinet, S. Douté, B. Schmitt, C. Sotin, E. Hauber, H. Hoffmann, R. Jaumann, U. Keller, R. Arvidson, J. F. Mustard, T. Duxbury, F. Forget, G. Neukum, Global mineralogical and aqueous mars history derived from OMEGA/Mars Express data. *Science* **312**, 400–404 (2006).

113. D. M. Pyle, T. A. Mather, Halogens in igneous processes and their fluxes to the atmosphere and oceans from volcanic activity: A review. *Chem. Geol.* **263**, 110–121 (2009).

114. M. Y. Zolotov, Martian volcanic gases: Are they Terrestrial-like? *XXXIV Lunar and Planet Sci* (Lunar and Planetary Institute, Houston, Abs. No. 1795, 2003).

115. H. Nair, M. Allen, A. D. Anbar, Y. L. Yung, R. T. Clancy, New Collective Author, A photochemical model of the Martian atmosphere. *Icarus* **111**, 124–150 (1994).

116. V. A. Krasnopolsky, Uniqueness of a solution of a steady state photochemical problem: Applications to Mars. *J. Geophys. Res. Planets* **100**, 3263–3276 (1995).

117. A. S. Braude, F. Montmessin, K. S. Olsen, A. Trokhimovskiy, O. I. Korablev, F. Lefèvre, A. A. Fedorova, J. Alday, L. Baggio, A. Irbah, G. Lacombe, F. Forget, E. Millour, C. F. Wilson, A. Patrakeev, A. Shakun, No detection of SO_2, H_2S, or OCS in the atmosphere of Mars from the first two Martian years of observations from TGO/ACS. *Astron. Astrophys.* **658**, A86 (2022).

118. Y. A. Taran, M. E. Zelenski, Systematics of water isotopic composition and chlorine content in arc-volcanic gases. *Geol. Soc. Lond. Spec. Publ.* **410**, 237–262 (2015).

119. R. E. Stoiber, W. I. Rose, The geochemistry of Central American volcanic gas condensates. *Geol. Soc. Am. Bull.* **81**, 2891–2912 (1970).

120. W. F. Giggenbach, Variations in the carbon, sulfur and chlorine contents of volcanic gas discharges from White Island, New Zealand. *Bull. Volcanol.* **39**, 15–27 (1975).

121. R. Faivre-Pierret, D. Martin, J. C. Sabroux, Contribution des sondes aérologiques motorisées à l'etude de la physico-chimie des panaches volcaniques. *Bull. Volcanol.* **43**, 473–485 (1980).

122. J. Hirabayashi, J. Ossaka, T. Ozawa, Relationship between volcanic activity and chemical composition of volcanic gases—A case study on the Sakurajima Volcano. *Geochem. J.* **16**, 11–21 (1982).

123. L. L. Malinconico, Fluctuations in SO_2 emission during recent eruptions of Etna. *Nature* **278**, 43–45 (1979).

124. L. L. Malinconico, On the variation of SO_2 emission from volcanoes. *J. Volcanol. Geotherm. Res.* **33**, 231–237 (1987).

125. T. P. Fischer, G. Chiodini, "Chapter 45—Volcanic, magmatic and hydrothermal gases," *The Encyclopedia of Volcanoes*, H. Sigurdsson, Eds. (Academic Press, ed. 2, 2015), pp. 779–797.

126. W. I. Rose, R. E. Stoiber, L. L. Malinconico, "Eruptive gas compositions and fluxes of explosive volcanoes: Budget of S and Cl emitted from Fuego volcano, Guatemala." *Andesites: Orogenic Andesites and Related Rocks*, R. S. Thorpe, Eds., (John Wiley and Sons, 1982), pp. 669–676.

127. E. Ilyinskaya, R. S. Martin, C. Oppenheimer, Aerosol formation in basaltic lava fountaining: Eyjafjallajökull volcano, Iceland. *J. Geophys. Res. Atmos.* **117**, D00U27 (2012).

128. X. Gutiérrez, F. Schiavi, H. Keppler, The adsorption of HCl on volcanic ash. *Earth Planet. Sci. Lett.* **438**, 66–74 (2016).

129. M. Ohno, M. Utsugi, T. Mori, I. Kita, T. Kagiyama, Y. Tanaka, Temporal variation in the chemical composition (HCl/SO_2) of volcanic gas associated with the volcanic activity of Aso Volcano, Japan. *Earth Planets Space* **65**, e1–e4 (2013).
